# Supplementary material for: Secondary analyses to test the impact on inequalities and uptake of the schools-based human papillomavirus (HPV) vaccination programme by stage of implementation of a new consent policy in the south-west of England
Source: BMJ Open. 2021 Jul 7;11(7):e044980. doi: 10.1136/bmjopen-2020-044980 (PMC8264897; doi:10.1136/bmjopen-2020-044980)
Supplement: Supplementary data [file bmjopen-2020-044980supp001.pdf]

Supplementary material 1. Pathway of new consent procedures

Stage One

Parental written consent

- Consent forms sent home via school to parents
- School collate forms for the vaccination session
- Young women with signed consent forms receive the HPV vaccine

Stage Two

Parental verbal consent & adolescent self-consent

- For young women without signed consent forms, parents telephoned for opportunity to verbally consent
- Young women asked whether discussion about vaccination had taken place at home
- Young women assessed for self-consent by immunisation team

Stage Three

Community catch-up clinics & family practice

- Unvaccinated young women are provided with written information about community catch-up clinics
- Some young women may also choose to be vaccinated in the family practice setting
