# Supplementary material for: Secondary analyses to test the impact on inequalities and uptake of the schools-based human papillomavirus (HPV) vaccination programme by stage of implementation of a new consent policy in the south-west of England
Source: BMJ Open. 2021 Jul 7;11(7):e044980. doi: 10.1136/bmjopen-2020-044980 (PMC8264897; doi:10.1136/bmjopen-2020-044980)
Supplement: Author's manuscript [file bmjopen-2020-044980.draft_revisions.pdf]

BMJ Open

BMJ Open is committed to open peer review. As part of this commitment we make the peer review history of every article we publish publicly available.

When an article is published we post the peer reviewers' comments and the authors' responses online. We also post the versions of the paper that were used during peer review. These are the versions that the peer review comments apply to.

The versions of the paper that follow are the versions that were submitted during the peer review process. They are not the versions of record or the final published versions. They should not be cited or distributed as the published version of this manuscript.

BMJ Open is an open access journal and the full, final, typeset and author-corrected version of record of the manuscript is available on our site with no access controls, subscription charges or pay-per-view fees (http://bmjopen.bmj.com).

If you have any questions on BMJ Open's open peer review process please email info.bmjopen@bmj.com

BMJ Open

Stages to increase uptake of the schools-based Human Papillomavirus (HPV) vaccination programme: Are they successful and do they decrease inequalities in uptake?

Journal:	*BMJ Open*
Manuscript ID	bmjopen-2020-044980
Article Type:	Original research
Date Submitted by the Author:	27-Sep-2020

Complete List of Authors:	Fisher, Harriet; University of Bristol, Bristol Medical School<br>Evans, Karen; Sirona Care and Health CIC<br>Reynolds, Rosy; University of Bristol, Bristol Medical School<br>Yates, Julie; Public Health England<br>Roderick, Marion; University Hospitals Bristol and Weston NHS Foundation Trust<br>Ferrie, Jo; Public Health England<br>Macleod, John; University of Bristol, School of Social and Community Medicine<br>Hickman, Matthew; University of Bristol, School of Social and Community Medicine<br>Audrey, Suzanne; University of Bristol, Schoolof Social and Community Medicine

Keywords:	PUBLIC HEALTH, INFECTIOUS DISEASES, Community child health < PAEDIATRICS

SCHOLARONE™
Manuscripts

I, the Submitting Author has the right to grant and does grant on behalf of all authors of the Work (as defined in the below author licence), an exclusive licence and/or a non-exclusive licence for contributions from authors who are: i) UK Crown employees; ii) where BMJ has agreed a CC-BY licence shall apply, and/or iii) in accordance with the terms applicable for US Federal Government officers or employees acting as part of their official duties; on a worldwide, perpetual, irrevocable, royalty-free basis to BMJ Publishing Group Ltd ("BMJ") its licensees and where the relevant Journal is co-owned by BMJ to the co-owners of the Journal, to publish the Work in this journal and any other BMJ products and to exploit all rights, as set out in our licence.

The Submitting Author accepts and understands that any supply made under these terms is made by BMJ to the Submitting Author unless you are acting as an employee on behalf of your employer or a postgraduate student of an affiliated institution which is paying any applicable article publishing charge ("APC") for Open Access articles. Where the Submitting Author wishes to make the Work available on an Open Access basis (and intends to pay the relevant APC), the terms of reuse of such Open Access shall be governed by a Creative Commons licence – details of these licences and which Creative Commons licence will apply to this Work are set out in our licence referred to above.

Other than as permitted in any relevant BMJ Author's Self Archiving Policies, I confirm this Work has not been accepted for publication elsewhere, is not being considered for publication elsewhere and does not duplicate material already published. I confirm all authors consent to publication of this Work and authorise the granting of this licence.

For peer review only

TITLE PAGE

Title: Stages to increase uptake of the schools-based Human Papillomavirus (HPV) vaccination programme: Are they successful and do they decrease inequalities in uptake

Dr Harriet Fisher[1]

Ms Karen Evans[2]

Dr Rosy Reynolds[1]

Dr Julie Yates[3]

Dr Marion Roderick[5]

Ms Jo Ferrie[3]

Professor John Macleod[1]

Professor Matthew Hickman[1]

Dr Suzanne Audrey[1]

[1] Population Health Sciences, Bristol Medical School, University of Bristol, Bristol, UK

[2] Head of School Nursing (Sirona) and Specialist Nursing Services, Bristol and South Gloucestershire, UK

[3] Screening and Immunisations South West, Public Health England, Bristol, UK

[4] Bristol Biomedical Research Centre and NIHR CLAHRC West, Bristol, UK

[5] Department of Paediatric Immunology & Infectious Diseases, Bristol Children's Hospital, Bristol, UK

Corresponding author:

Dr Harriet Fisher

Population Health Sciences, Bristol Medical School

University of Bristol

Canynge Hall

39 Whatley Road

Bristol, BS8 2PS

For peer review only

Email: Harriet.Fisher@bristol.ac.uk

Telephone: 0117 928 7223

Word count (main text): 3,179

Keywords: HPV vaccination programme; adolescents; consent; process evaluation; inequalities

For peer review only

For peer review only - http://bmjopen.bmj.com/site/about/guidelines.xhtml

ABSTRACT

Objectives: To test the impact on inequalities and uptake of the schools-based human papillomavirus (HPV) vaccination programme by stage of implementation of a new policy providing additional opportunities to consent.

Setting: Two local authorities in the South West of England.

Participants: Young women (n=7,129) routinely eligible for HPV vaccination aged 12-13 years during the intervention period (2017/18 to 2018/19 programme years).

Interventions: Local policy change that included additional opportunities to provide consent (parental verbal consent and adolescent self-consent).

Outcomes: Secondary analyses on cross-sectional intervention data were undertaken to examine uptake by: (i) receipt of parental written consent forms, and; (ii) percentage of unvaccinated young women by stage of implementation.

Results: During the intervention period, 6,341 (89.0%) eligible young women initiated the HPV vaccination series. Parental written consent forms were less likely to be returned where young women attended alternative education provider settings ($p<0.001$), belonged to Non-White British ethnic groups ($p<0.01$) or more deprived quintiles ($p<0.001$). Implementation of parental verbal consent and adolescent self-consent reduced the percentage of unvaccinated young women from 21.3% to 16.5% (risk difference: 4.8%). The effect was greater for young women belonging to the most deprived compared to the least deprived quintile (risk difference: 7.4% vs. 2.3%, $p<0.001$), and for young women classified as Unknown ethnic category compared to White British young women (6.7% vs. 4.2%, $p<0.001$). No difference was found for Non-White British young women (5.4%, $p<0.21$).

Conclusions: Allowing parents to consent verbally and adolescent-self consent overcame some of the barriers to vaccination of young women belonging to families less likely to respond to paper-based methods of gaining consent.

Word count: 262

Keywords: HPV vaccination programme; adolescents; consent; process evaluation; inequalities

Name of the registry: ISRCTN registry

Trial registration number: 49086105

Date of registration: 12/01/2018

For peer review only - http://bmjopen.bmj.com/site/about/guidelines.xhtml

URL of trial registry record: www.isrctn.com/ISRCTN49086105

ARTICLE SUMMARY

Strengths and limitations of the study

This study utilises routinely collected data to examines the impact of local policy changes on inequalities in uptake of the HPV vaccination programme

The intervention period (programme years 2017/18 – 2018/19) was relatively short

Missing data on ethnicity could change the direction or size of the corresponding Odds Ratios

INTRODUCTION

The Human Papillomavirus (HPV) vaccine currently used in England protects against infection from high-risk HPV types 16 and 18 which cause cancers affecting the cervix, vulva, vagina, penis, anus, and oral cavity. The vaccine also protects against types 6 and 11 which cause 90% of genital warts. High coverage of the English HPV vaccination programme for young women aged 12 to 13 years has been achieved. Recent evidence highlights potential for HPV vaccination programmes to substantially reduce the incidence of cervical cancer [1, 2]. Based on emerging evidence for cost-effectiveness, in 2019/20 the HPV vaccination programme was expanded to include young men aged 12 to 13 years.

Despite generally good coverage, without concerted efforts to address lower uptake amongst some populations, pre-existing disparities in the incidence of cervical cancer by ethnicity and deprivation may increase [3-5]. We previously identified lower uptake by area and amongst some population groups, including minority ethnic groups [6]. Our research in schools with lower uptake showed complex socio-cultural factors can influence whether young women are vaccinated [7] and the requirement for written parental consent may act as a barrier to some young women receiving the HPV vaccine [8].

In the United Kingdom (UK), the legal framework allows young people to be vaccinated without parental consent provided they are deemed 'Gillick competent' [9] (e.g. they have sufficient maturity and intelligence to understand the nature and implications of the treatment). But findings from an evidence synthesis showed the implementation of adolescent self-consent procedures could be prevented by

For peer review only - http://bmjopen.bmj.com/site/about/guidelines.xhtml

local policies which favoured written parental consent, and precedence given to professionals' concerns about their reputations and relationships with parents [10].

A local authority is an organisation officially responsible for all public services and facilities in a particular area. To address concerns about lower uptake in two local authority areas in the south-west of England, a new policy (including parental verbal consent and adolescent self-consent) has been implemented [9]. Implementation of the new consent procedures in one of the intervention areas appeared to improve uptake in contrast to trends of decreasing uptake among matched local authorities [11]. However, no evidence for an absolute increase, or reduction in inequalities by deprivation and ethnicity was found.

The aim of the current study is to further examine the impact of the consent procedures on HPV vaccine uptake. Specifically, we describe:

(i) Receipt of parental written consent forms by school category, ethnicity and deprivation quintile

(ii) Unvaccinated young women by stage of implementation of consent procedures, and by school category, ethnicity and deprivation quintile

METHODS

This cross-sectional study was undertaken when the English vaccination programme was delivered routinely to young women only. Details of the evaluation and changes to the new local policy are provided in a published protocol [12].

The new local policy (including consent procedures)

In brief, previously only young women who had returned a written parental consent form, indicating the parent or carer is willing for their daughter to receive the HPV vaccine, were administered the vaccine in the school setting. Under the new arrangements, all young women eligible for the vaccination, including those whose parents have provided written refusal, are asked to attend the session by the immunisation team. For those young women who do not have a returned parental written consent form, the immunisation nurse attempts to gain parental verbal consent over the telephone. If the parent cannot

For peer review only - http://bmjopen.bmj.com/site/about/guidelines.xhtml

be contacted and the young woman expresses willingness to be vaccinated, the immunisation nurse assesses the young woman's competence and if they are deemed competent the young women will receive the vaccine. All young women who do not receive the vaccine on the day, are given information about alternative options to receive the vaccination, such as through their family doctor or community-based clinics run by the immunisation team (Supplementary material 1). Parents are not routinely contacted again by the immunisation team.

Patient and public involvement

The Bristol Young Person's Advisory Group (YPAG) took part in preliminary discussions about self-consent procedures, at which a group of 11 young people discussed self-consent for vaccination of young people who are of secondary school age. Following this discussion, all voted in favour of self-consent preferably with parents being informed. The young people were overall positive about the documents which they thought they provided the right amount of information clearly. As a result of the feedback, some changes to the wording of the information and formatting were made. Members of the Bristol Youth Council were approached to participate in the study during recruitment. This resulted in an opportunity to obtain feedback on a proposal developed from the findings of the current study.

During the study, the study researcher was also invited to deliver information sessions about the HPV vaccine to Year 7 students. The study researcher also led PHSE lessons where Year 8 students were able to find out about research and encouraged to debate issues around adolescent self-consent. Study findings are being shared with the YPAG and the Bristol City Youth Council at meetings to mark the end of the study.

Population

Two local authorities implementing the new consent procedures for the HPV vaccination programme in the south-west of England provided data. Records relating to young women eligible (born between 1st September 2004 and 31st August 2006) for vaccination during the two-year intervention period (programme years 2017/18-2018/19) and who were registered with a general practice within the local authority boundaries were retrieved in July 2019 from the Child Health Information System.

For peer review only - http://bmjopen.bmj.com/site/about/guidelines.xhtml

Data extraction from the Child Health Information System

Prior to study commencement, permission to access an anonymised data extract was gained from the relevant organisations with responsibility for the data. In the UK, the Child Health Information System holds demographic and vaccination-related records for each young person registered with a family doctor which is a statutory requirement. The following data fields were extracted from records of the eligible population: (i) partial date of birth; (ii) partial postcode; (iii) ethnicity; (iv) dates and location HPV vaccination administered, and; (v) name and corresponding identifying code of school.

School identifying code was used to assign local authority responsible for delivery of the HPV vaccine. Partial date of birth was used to allocate programme year the young woman was eligible to receive the HPV vaccine. Categories of school types were applied to each record: (i) comprehensive, non-fee-paying; (ii) private, fee-paying, and; (iii) alternative education provider, which included pupil referral units, young offender units, hospital education service, specialist schools for students with significant additional needs and young women educated at home.

Individual records were classed as 'received HPV vaccine' if there was a record of at least one dose administered within the corresponding programme year the young woman was eligible. Postcodes from individual records were linked to the corresponding Lower Super Output Area (LSOA). Deprivation score was assigned using the Index of Multiple Deprivation 2019 (a statistic on relative deprivation in small areas of England) [13] and analysed as quintiles. Due to small numbers, ethnicity was grouped as follows: (i) White British; (ii) Non-White British, and; (iii) Unknown.

Records were excluded if the relevant school identifying code was missing or invalid. Absence of recorded ethnicity was considered likely Missing Not At Random as absence of ethnicity data was associated with the outcome, school and deprivation variables. A complete case approach where records were excluded on the basis of missing ethnicity is not recommended [14]. Instead, the 'Unknown' category was assigned to missing ethnicity data to minimise the risk of bias.

Data extraction from the immunisation team's records of consent

For peer review only - http://bmjopen.bmj.com/site/about/guidelines.xhtml

Additional data was sought from the school immunisation team's electronic and paper-based records relating to vaccination consent during the intervention period. This included: (i) return of parental consent forms ('yes' or 'no') and; (ii) stage of implementation of consent procedures ('Stage One: parent written consent only', 'Stage Two: parent verbal consent & adolescent self-consent', and 'Stage Three: community catch up clinics and family practice settings').

Data linkage

The data extracted from the immunisation team's records were linked to the Child Health Information System using deterministic data linkage methods by a member of staff at Health Intelligence. An anonymised version of the data extract was securely transferred to researchers at the University of Bristol.

ANALYSIS

Return of parental consent forms

Logistic univariable analyses and likelihood ratio tests were performed to explore factors associated with return of parental consent form. The following explanatory variables for analysis were selected *a priori*: school category, ethnicity and deprivation quintile. A multivariable logistic regression model was developed. We used cluster-robust errors in the final model to allow for the possibility of clustering within schools.

Unvaccinated young women by stage of implementation of consent procedures

To describe the decrease in unvaccinated young women at each stage of implementation of the consent procedures, we calculated risk differences (difference in two proportions) with 95% confidence intervals. We considered the following risk differences by: (i) percentage of young women unvaccinated during '*Stage One: parent written consent only*' minus percentage of young women unvaccinated during '*Stage One' and 'Stage Two: parent verbal consent & adolescent self-consent*', and; (ii) percentage of young women unvaccinated during '*Stage One'* minus percentage of young women unvaccinated during *'Stage One', 'Stage Two' and 'Stage Three: community catch up clinics and family practices'*).

For peer review only - http://bmjopen.bmj.com/site/about/guidelines.xhtml

To show whether there was an unintended increase or reduction in health inequalities, we compared the risk differences and corresponding p-values by school category, ethnic group, and deprivation quintile – comparing with a baseline category in each case.

Analyses were undertaken using the Stata statistical package, release 15 (Stata Corp, College Station, TX).

RESULTS

Data were extracted relating to 7,549 young women eligible for vaccination during the intervention period (programme years 2017/18 to 2018/19). Of these, 420 (5.6%) were excluded on the basis that the school data was missing or invalid.

Of the cohort retained for analysis (n=7,129), the majority of vaccine eligible young women were resident in local authority one (4,516, 63.4%), attended comprehensive, non-fee paying schools (6,350, 89.1%), and were classified as belonging to a White British ethnic group (4,888, 68.6%). Of young women eligible for vaccination, 6,341 (89.0%) were recorded to have received the HPV vaccine during the programme year they were eligible. Parental consent forms were recorded as being unreturned (comprising active non-consent and passive non-consent) for 1,555 (16.2%) of eligible young women (Table 1).

Return of parental consent forms

After adjusting for school category, ethnicity, deprivation, there was strong evidence that the parental consent form not being returned was related to attending an alternative education provider setting (adjusted Odds Ratio (aOR): 5.54, 95% CI: 3.80-8.09, $p<0.001$), or belonging to a Non-White British (aOR: 1.34, 95% CI: 1.06-1.70, $p<0.01$) or Unknown ethnicity category (aOR: 2.41, 95% CI: 2.09-2.78, $p<0.001$). There was also evidence for a relationship with level of deprivation. For example, young women belonging to the most deprived quintile had at least double the odds of having a record of unreturned consent form (aOR: 2.54, 95% CI: 2.03-3.18, $p<0.001$) compared to those from the least deprived quintile (Table 2).

For peer review only - http://bmjopen.bmj.com/site/about/guidelines.xhtml

Percentage of young women unvaccinated by stage of implementation of consent procedures

Following implementation of Stage One (parental written consent) of the consent procedures, 1,519 (21.3%) of young women were unvaccinated. At Stage Two (parental verbal consent and adolescent self-consent), this reduced to 1,173 (16.5%) unvaccinated young women. With the inclusion of Stage Three (community catch up clinics and family practice settings), there remained 788 (11.1%) unvaccinated young women during the study period (Table 4). Not all parents could be contacted by the immunisation team on the day of the vaccination session (n=362). These parents are not routinely contacted again by the immunisation team (data not shown).

The percentage of unvaccinated young women varied by school category at different stages of implementation of the consent procedures. For example, at Stage One (parental written consent only), 20.6% of young women who attended mainstream comprehensive, non-fee-paying schools were unvaccinated, in comparison to 50.8% who attended alternative education provider settings. At Stage Two (parental verbal consent and adolescent self-consent), there was no evidence for narrowing of this gap (p=0.27). However after Stage Three (community catch-up clinics and family practice settings), the decrease in unvaccinated young women was greater for those that attended alternative education provider settings, compared to those who attended comprehensive, non-fee paying schools (accumulative risk difference: 23.7% vs. 10.2%, p<0.001) (Figure 1 & Supplementary Material 2).

There were also differences by ethnicity. At Stage One (parental written consent only), 15.8% of White British young women were unvaccinated, in comparison to 26.7% of Non-White British young women and 35.7% belonging to the Unknown ethnic category. Following implementation of Stage Two (parental verbal consent and adolescent self-consent), the percentage unvaccinated decreased at a greater rate in young women classified as 'Unknown' ethnic category compared to White British young women (risk difference: 6.7% vs 4.2%, p<0.001), but no difference was observed for Non-White British young women (p=0.21). There was evidence for a difference with the inclusion of Stage Three for Non-White British women (accumulative risk difference: 12.8% vs. 8.9%, p=0.01) and Unknown ethnicity (accumulative risk difference: 13.4% vs. 8.9%, p<0.001) (Figure 1 & Supplementary Material 2).

For peer review only - http://bmjopen.bmj.com/site/about/guidelines.xhtml

Inequalities in the percentage of unvaccinated young women by deprivation were attenuated by each stage of implementation of the policy. At Stage One (parental written consent only), 14.2% young women in the least deprived quintile were unvaccinated in comparison to 29.8% of young women in the most deprived quintile. Subsequent to Stage Two (parental verbal consent and adolescent self-consent), the percentage of unvaccinated young women decreased at a greater rate for those belonging to the most deprived quintile compared to the least deprived quintile (risk difference: 7.4% vs. 2.3%, p<0.001). A similar pattern was observed following implementation of Stage Three (community catch-up clinics and family practice settings) (overall risk difference: 13.8% vs. 5.8%, p<0.001) (Figure 1 & Supplementary Material 2).

DISCUSSION

Previous research has shown that young women from more disadvantaged backgrounds are more likely to be affected by cervical cancer [15, 16], and less likely to receive the HPV vaccine [6]. The findings from this study show that some of the barriers to young women being vaccinated were overcome through the implementation of new local policy, which included parental verbal consent and adolescent self-consent in the school setting. There is promising evidence that the additional steps have the potential to reduce existing inequalities in uptake among young women living in more deprived areas. Importantly, this study showed that these young women are less likely to engage with consent procedures that rely on paper-based methods.

The provision of the HPV vaccine in community settings helped reduce substantial inequalities in uptake among young women educated in alternative education provider settings. Although they comprise a small proportion of the overall vaccine-eligible population, they are a vulnerable population with substantially lower uptake which requires addressing. Barriers to uptake of the HPV vaccination programme by these vulnerable young women in these settings are multifaceted, and may be related to lower attendance, complex behavioural and physical health needs, perceptions of safety of the vaccine due to interactions with medical treatment, or beliefs related to sexual behaviours of young people with learning disabilities.

For peer review only

For peer review only - http://bmjopen.bmj.com/site/about/guidelines.xhtml

As a result of the new schools-based consent procedures, an additional 347 young women (4.8%) received the HPV vaccine in the school setting during the intervention period. As the majority of parents who could be contacted provided verbal consent, the data imply that the absence of a signed parental consent form cannot be assumed to mean the parent does not want their daughter to have the vaccine. Not all parents could be contacted by the immunisation team on the day of the vaccination session. Provision of additional resources to contact families ahead of the vaccination session could help reduce the proportion of families who are not contacted and help ensure that their daughter receives the HPV vaccine if they wish.

Relative to parental verbal consent, adolescent self-consent occurred infrequently. Barriers to implementation of adolescent self-consent procedures may relate to the age of vaccine-eligible young women (12 to 13 years old). Separate analyses of qualitative data from this study will report the acceptability of different methods of obtaining consent from the perspectives of young women, parents and professionals involved with the HPV vaccination programme.

Public Health England have recently issued updated guidance for healthcare professionals related to the new universal HPV vaccination programme [17]. This supports the use of parent verbal consent and adolescent self-consent as strategies to maximise uptake and reduce catch-up sessions. They cite further benefit of inclusion of young people whose parents may have difficulties in completing the consent due to language or literacy issues. The findings from this study provide evidence that strategies incorporating parent verbal consent could help young women belonging to 'harder-to-reach' families receive the HPV vaccine. These recommendations may also be applicable to other schools-based vaccination programmes, including the influenza vaccination programme offered to primary school aged children where similar patterns in forms returns have been reported [18].

Strengths and limitations

The study has some strengths. This is the first study to examine how new local polices for the HPV vaccination programme are implemented, and the impact on health inequalities among more deprived populations and young women belonging to minority ethnicity groups. Our study utilised routinely collected data related to vaccination status eliminating the risk of recall and selection bias. The data

For peer review only

relate to vaccinations delivered in school and community settings to all young women eligible for routine HPV vaccination during the study period. As such, our results correspond to an almost complete population.

There are some limitations. The data related to implementation of new consent procedures in a geographically distinct area in the south-west of England. The findings therefore may not be applicable to local authorities that are implementing new consent procedures in schools-based vaccination programmes elsewhere in the United Kingdom. The findings may also not translate to other adolescent vaccination programmes delivered in countries where cultural differences may influence the acceptability of parental verbal consent and adolescent self-consent procedures in school-setting.

As the study relied on routinely collected information, we did not have access to individual-level measures of socioeconomic status and relied on area-based measures of deprivation. Our study findings may therefore be subject to ecological fallacy.

An issue, common to all routinely collected data, is the possibility of data input errors and missing data. To minimise bias from inclusion of this data, we excluded almost 5% of the data as the information related to school was out-of-date. Overall, our dataset identified 8% of young women belonging to a minority ethnic group. This compares with nationally reported figures indicating 30% of young people attending secondary schools in the intervention areas belong to a non-White ethnic group [19]. Missing ethnicity data (23%) relating to young women who were born outside the local authority boundaries could change the direction or size of aORs corresponding to ethnicity.

Conclusions

Introducing further steps to the consent procedures – allowing parents to consent verbally and adolescent-self consent – overcame some of the barriers to vaccination of young women belonging to families less likely to respond to paper-based methods of gaining consent.

For peer review only - http://bmjopen.bmj.com/site/about/guidelines.xhtml

REFERENCES

1. Drolet, M., et al., Population-level impact and herd effects following the introduction of human papillomavirus vaccination programmes: updated systematic review and meta-analysis. The Lancet, 2019. **394**(10197): p. 497-509.

2. Simms, K., et al., Impact of scaled up human papillomavirus vaccination and cervical screening and the potential for global elimination of cervical cancer in 181 countries, 2020–99: a modelling study. The Lancet Oncology, 2019. **20**(3): p. 394-407.

3. Johnson, H., et al., Effect of HPV vaccination and cervical cancer screening in England by ethnicity: a modelling study. The Lancet Public Health, 2018. **3**(1): p. e44-e51.

4. Baker, D. and E. Middleton, Cervical screening and health inequality in England in the 1990s. 2003. **57**(6): p. 417-423.

5. Shack, L., et al., Variation in incidence of breast, lung and cervical cancer and malignant melanoma of skin by socioeconomic group in England. BMC Cancer, 2008. **8**(1): p. 271.

6. Fisher, H., et al., Examining inequalities in the uptake of the school-based HPV vaccination programme in England: a retrospective cohort study. Journal of Public Health, 2013. **36**(1): p. 36-45.

7. Batista Ferrer, H., et al., Barriers and facilitators to uptake of the school-based HPV vaccination programme in an ethnically diverse group of young women. Journal of Public Health, 2016. **38**(3): p. 569-577.

8. Batista Ferrer, H., Factors influencing the uptake of the Human Papillomavirus (HPV) vaccination programme. Thesis (Ph.D.), 2014.

9. Gillick V . West Norfolk and Wisbech Area Health Authority and Department of Health and Social Security. 1985, House of Lords: London.

10. Fisher, H., et al., Barriers and enablers to adolescent self-consent for vaccination: A mixed-methods evidence synthesis. Vaccine, 2019. **37**(3): p. 417-429.

11. Fisher, H., et al., Impact of new consent procedures on uptake of the schools-based Human Papillomavirus (HPV) vaccination programme: A quasi experimental study. Journal of Public Health, 2020, Accepted/In Press.

12. Audrey, S., et al., Impact and acceptability of self-consent procedures for the school-based human papillomavirus vaccine: a mixed-methods study protocol. 2018. **8**(3): p. e021321.

13. English indices of deprivation, 2019. Available from: https://www.gov.uk/government/statistics/english-indices-of-deprivation-2019.

14. Hughes, R., et al., Accounting for missing data in statistical analyses: multiple imputation is not always the answer. International Journal of Epidemiology, 2019. **48**(4): p. 1294-1304.

15. Shack, L., et al., Variation in incidence of breast, lung and cervical cancer and malignant melanoma of skin by socioeconomic group in England. BMC Cancer, 2008. **8**: p. 271.

16. Cancer Research UK and National Cancer Intelligence Network, 2014. Cancer by deprivation in England: Incidence, 1996-2010, Mortality, 1997-2011. 2014, NCIN: London.

17. Public Health England, HPV vaccination: Information for healthcare practitioners, 2019, Available from: https://assets.publishing.service.gov.uk/government/uploads/system/uploads/attachment_data/file/813014/PHE_HPV_universal_programme_guidance.pdf.

For peer review only

For peer review only - http://bmjopen.bmj.com/site/about/guidelines.xhtml

18. Christensen, H., et al., Influence of commissioned provider type and deprivation score on uptake of the childhood flu immunization. Journal of Public Health, 2019.

19. Department for Education, Schools, pupils and their characteristics: January 2018. Available from: https://www.gov.uk/government/statistics/schools-pupils-and-their-characteristics-january-2018.

DECLARATIONS

Consent & ethical considerations

Research Ethics Committee approval is not required for evaluations that are designed solely to define or judge current care and measure a current service without reference to a standard. This evaluation involves the retrospective analysis of an anonymised dataset comprising data routinely collated by the immunisation teams and the Child Health Information Services as part of the HPV vaccination programme. The study did not involve the collection of additional data. Research Ethics Committee approval was therefore not required. However, permission for the research team at the University of Bristol to receive an anonymised dataset was sought from the organisations assigned as data custodians for the data (Sirona Health and Care and In Health Intelligence).

Patient consent

Not required.

Data sharing statement

As the analysis was undertaken on routine data acquired from an external source, the study authors assured the data custodians that would the dataset would be treated as confidential and would not be shared.

Competing interests

The authors have no competing interests to declare.

Funding

This work is funded by the National Institute for Health Research's Research for Patient Benefit (NIHR RfPB) programme (project number PB-PG-0416-20013). The views and opinions expressed therein are

For peer review only - http://bmjopen.bmj.com/site/about/guidelines.xhtml

For peer review only

those of the authors and do not necessarily reflect those of the NIHR RfPB Programme or the Department of Health.

Contributorship statement

SA, HF, MH, KE, MB, JF & MR were involved in the conception and design of the research. SA is principal investigator; HF is study manager and undertook data cleaning, analysed the data, and drafted the manuscript; MB facilitated PPI; JF, JY and KE developed and advised on the new consent procedures and the HPV vaccination process; MR advised on local immunisation strategy; JM advised on health inequalities and MH and RR advised on statistical methods. All authors have made substantial contributions to interpreting the data, revising it for important intellectual content, and have given approval of the final version to be submitted.

Acknowledgements

The authors express their gratitude to staff at In Health Intelligence for data linkage and extracting the data from the Child Health Information System, and to the immunisation and administrative teams at Sirona Healthcare who facilitated access to the immunisation consent records as part of this study.

The study is partly supported by the NIHR Health Protection Research Unit in Evaluation of Interventions at University of Bristol. The work was undertaken with the support of The Centre for the Development and Evaluation of Complex Interventions for Public Health Improvement (DECIPHer), a UKCRC Public Health Research Centre of Excellence. Joint funding (MR/KO232331/1) from the British Heart Foundation, Cancer Research UK, Economic and Social Research Council, Medical Research Council, the Welsh Government and the Wellcome Trust, under the auspices of the UK Clinical Research Collaboration, is gratefully acknowledged.

For peer review only - http://bmjopen.bmj.com/site/about/guidelines.xhtml

Table 1. Descriptive summary of eligible cohort by vaccine receipt and return of parent consent form

	Eligible cohort n (%)	HPV vaccine received n (%)	Unreturned parent consent form n (%)
	7,129 (100.0)	6,341 (89.0)	1,155 (16.2)
Area-level			
Local Authority One	4,516 (63.4)	3,944 (87.3)	843 (18.7)
Local Authority Two	2,613 (36.7)	2,397 (91.7)	312 (11.9)
Programme year 2017/18	3,581 (50.2)	3,202 (89.4)	565 (15.8)
Programme year 2018/19	3,548 (49.8)	3,139 (88.5)	590 (16.6)
School category			
Comprehensive, non-fee-paying	6,350 (89.1)	5,690 (89.6)	992 (15.6)
Private, fee-paying	661 (9.3)	565 (85.5)	105 (15.9)
Alternative education providers	118 (1.7)	86 (72.9)	58 (49.2)
Individual-level			
Ethnicity			
White British	4,888 (68.6)	4,552 (93.1)	610 (12.5)
Non-White British	572 (8.0)	492 (86.0)	101 (17.7)
Unknown	1,669 (23.4)	1,297 (77.7)	444 (26.6)
Deprivation			
Least deprived	1,348 (18.9)	1,235 (91.6)	136 (10.1)
Quintile 2	1,379 (19.3)	1,277 (92.6)	149 (10.8)
Quintile 3	1,403 (19.7)	1,273 (90.7)	210 (15.0)
Quintile 4	1,396 (19.6)	1,203 (86.2)	292 (20.9)
Most deprived	1,421 (19.9)	1,194 (84.0)	338 (23.8)
Unknown	182 (3.6)	159 (87.4)	30 (16.5)

For peer review only

For peer review only - http://bmjopen.bmj.com/site/about/guidelines.xhtml

Table 2. Associations of unreturned parental consent form with school category, ethnicity & deprivation

	N (%)	Form not returned	OR (95% CI) †	p-value	aOR (95% CI) †	p-value
			-	-	-	-
School category		**n (%)**				
Comprehensive, non-fee-paying	6,350	992 (15.6)	-	-		
Private, fee-paying	661	105 (15.9)	1.02 (0.82-1.27)	0.86	1.12 (0.89-1.43)	0.34
Alternative education providers	118	58 (49.2)	5.22 (3.62-7.54)	<0.001	5.54 (3.80-8.09)	<0.001
Ethnicity						
White British	4,888	610 (12.5)	-	-	-	-
Non-White British	572	101 (17.7)	1.50 (1.19-1.89)	<0.01	1.34 (1.06-1.70)	0.01
Unknown	1,669	444 (26.6)	2.54 (2.21-2.92)	<0.001	2.41 (2.09-2.78)	<0.001
Deprivation						
Least deprived	1,279	136 (10.1)	-		-	
Quintile 2	1,324	149 (10.8)	1.08 (0.84-1.38)	0.54	1.09 (0.85-1.40)	0.48
Quintile 3	1,334	210 (15.0)	1.57 (1.25-1.97)	<0.001	1.57 (1.24-1.98)	<0.001
Quintile 4	1,305	292 (20.9)	2.36 (1.89-2.93)	<0.001	2.24 (1.79-2.81)	<0.001
Most deprived	1,347	338 (23.8)	2.78 (2.24-3.45)	<0.001	2.54 (2.03-3.18)	<0.001
Unknown	182	30 (16.4)	1.75 (1.14-2.70)	0.01	1.52 (0.97-2.37)	0.07

† Adjusted for ethnicity, deprivation, school category, and clustering by school; OR: Odds Ratio; CI: Confidence Intervals; aOR: Adjusted Odds Ratio;

For peer review only

For peer review only - http://bmjopen.bmj.com/site/about/guidelines.xhtml

Figure 1. Percentage of HPV vaccine eligible young women unvaccinated by stage of implementation of consent procedure

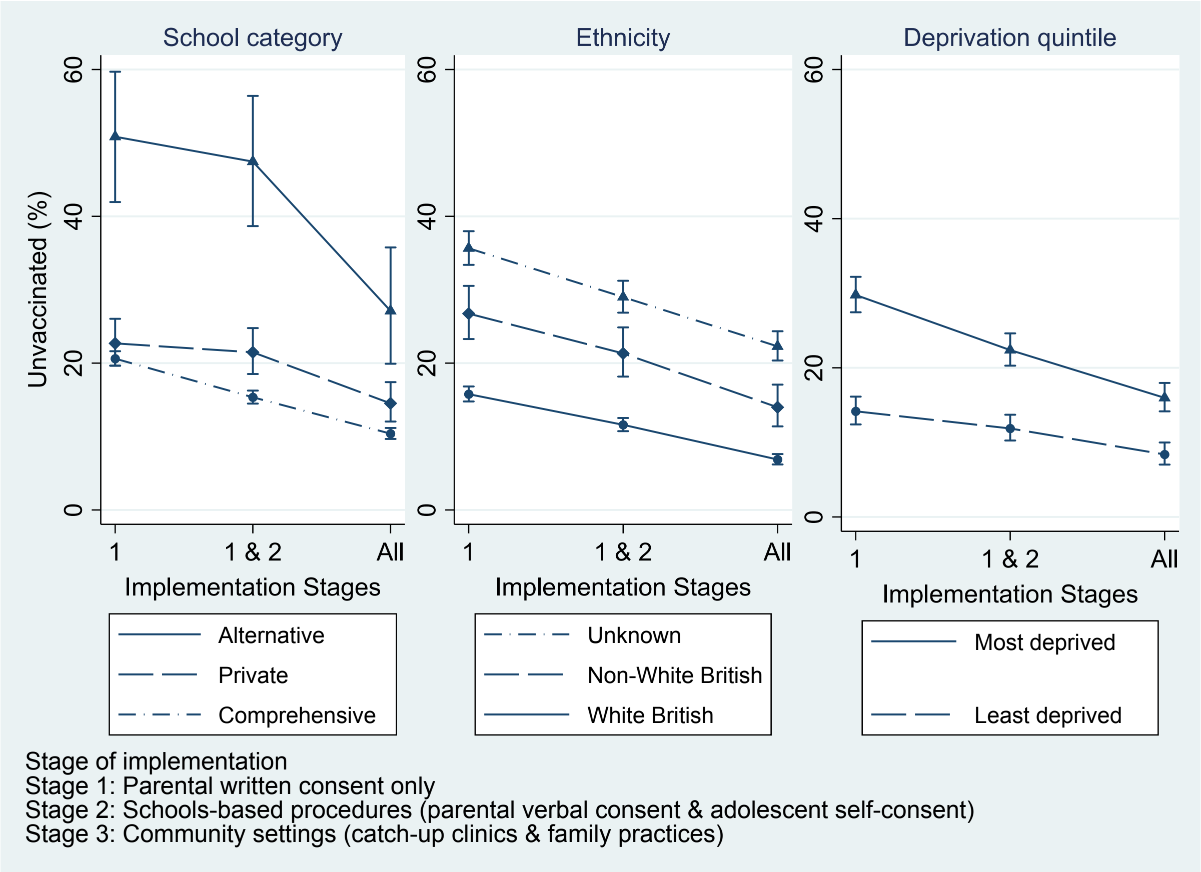

For peer review only - http://bmjopen.bmj.com/site/about/guidelines.xhtml

Supplementary material 1. Pathway of new consent procedures

Stage One

Parental written consent

- Consent forms sent home via school to parents
- School collate forms for the vaccination session
- Young women with signed consent forms receive the HPV vaccine

Stage Two

Parental verbal consent & adolescent self-consent

- For young women without signed consent forms, parents telephoned for opportunity to verbally consent
- Young women asked whether discussion about vaccination had taken place at home
- Young women assessed for self-consent by immunisation team

Stage Three

Community catch-up clinics & family practice

- Unvaccinated young women are provided with written information about community catch-up clinics
- Some young women may also choose to be vaccinated in the family practice setting

For peer review only

Supplementary material 2. Percentage of young women unvaccinated by implementation stage of consent process

		Stage 1†	Stages 1 & 2	Risk difference[1]	p-value[1]	Stages 1, 2 & 3	Risk difference[2]	p-value[2]
		n (%)	n (%)	n (%)		n (%)	n (%)	-
		1,519 (21.3)	1,173 (16.5)	347 (4.8)		788 (11.1)	731 (10.3)	
School category	**N (%)**	**% (95% CI)**	**% (95% CI)**	**% (95% CI)**	**-**	**% (95% CI)**	**% (95% CI)**	
Comprehensive, non-fee-paying	6,350	20.6 (19.6-21.6)	15.4 (14.5-16.3)	5.3 (4.7-5.8)	-	10.4 (9.7-11.2)	10.2 (9.5-11.0)	-
Private, fee-paying	661	22.7 (19.7-26.0)	21.5 (18.5-24.8)	1.2 (0.6-2.4)	<0.001	14.5 (12.0-17.4)	8.2 (6.3-10.5)	0.07
Alternative education providers	118	50.8 (41.9-59.7)	47.5 (38.7-56.4)	3.4 (1.3-8.7)	0.27	27.1 (19.9-35.8)	23.7 (16.9-32.3)	<0.001
Ethnicity								
White British	4,705	15.8 (14.8-16.8)	11.6 (10.7-12.5)	4.2 (3.6-4.8)	-	6.9 (6.2-7.6)	8.9 (8.1-9.7)	-
Non-White British	542	26.7 (23.3-30.5)	21.3 (18.2-24.9)	5.4 (3.8-7.6)	0.21	14.0 (11.4-17.1)	12.8 (10.3-15.8)	0.01
Unknown	1,503	35.7 (33.4-38.0)	29.0 (26.9-31.2)	6.7 (5.6-8.0)	<0.001	22.3 (20.4-24.3)	13.4 (11.8-15.1)	<0.001
Deprivation								
Least deprived	1,279	14.2 (12.4-16.1)	11.9 (10.3-13.7)	2.3 (1.6-3.3)	-	8.4 (7.0-10.0)	5.8 (4.7-7.2)	-
Quintile 2	1,324	14.9 (13.1-16.8)	11.2 (9.7-13.0)	3.6 (2.8-4.8)	0.04	7.4 (6.1-8.9)	7.5 (6.2-9.0)	0.08
Quintile 3	1,334	19.8 (17.8-22.0)	14.9 (13.1-16.9)	4.9 (3.9-6.2)	<0.001	9.3 (7.9-10.9)	10.5 (9.0-12.3)	<0.001
Quintile 4	1,305	27.1 (24.8-29.5)	20.8 (18.8-23.1)	6.2 (5.1-7.6)	<0.001	13.8 (12.1-15.7)	13.3 (11.6-15.1)	<0.001
Most deprived	1,347	29.8 (27.4-32.2)	22.4 (20.3-24.6)	7.4 (6.1-8.9)	<0.001	16.0 (14.2-18.0)	13.8 (12.1-15.7)	<0.001
Unknown		24.2 (18.5-30.9)	22.0 (16.6-28.5)	12.6 (8.6-18.2)	0.93	12.6 (8.5-18.3)	11.5 (7.6-17.1)	0.02

† Stage 1: Parental written consent only; Stage 2: Schools-based procedures (parental verbal consent & adolescent self-consent; Stage 3: Community settings (catch-up clinics & family practices); CI: Confidence intervals;
[1]Risk difference for stages 1 & 2 compared to stage 1; p-value for comparison with risk difference in baseline group.
[2]Risk difference for stages 1 & 2 & 3 compared to stage 1; p-value for comparison with risk difference in baseline group.

For peer review only

STROBE Statement—Checklist of items that should be included in reports of ***cross-sectional studies***

	Item No	Recommendation	Page No
**Title and abstract**	1	(*a*) Indicate the study's design with a commonly used term in the title or the abstract	3
		(*b*) Provide in the abstract an informative and balanced summary of what was done and what was found	3
**Introduction**			
Background/rationale	2	Explain the scientific background and rationale for the investigation being reported	4-5
Objectives	3	State specific objectives, including any prespecified hypotheses	5
**Methods**			
Study design	4	Present key elements of study design early in the paper	5
Setting	5	Describe the setting, locations, and relevant dates, including periods of recruitment, exposure, follow-up, and data collection	6-8
Participants	6	(*a*) Give the eligibility criteria, and the sources and methods of selection of participants	6-8
Variables	7	Clearly define all outcomes, exposures, predictors, potential confounders, and effect modifiers. Give diagnostic criteria, if applicable	6-8
Data sources/ measurement	8*	For each variable of interest, give sources of data and details of methods of assessment (measurement). Describe comparability of assessment methods if there is more than one group	6-8
Bias	9	Describe any efforts to address potential sources of bias	7
Study size	10	Explain how the study size was arrived at	7-9
Quantitative variables	11	Explain how quantitative variables were handled in the analyses. If applicable, describe which groupings were chosen and why	7-8
Statistical methods	12	(*a*) Describe all statistical methods, including those used to control for confounding	8-9
		(*b*) Describe any methods used to examine subgroups and interactions	8-9
		(*c*) Explain how missing data were addressed	7
		(*d*) If applicable, describe analytical methods taking account of sampling strategy	N/A
		(*e*) Describe any sensitivity analyses	N/A
**Results**			
Participants	13*	(a) Report numbers of individuals at each stage of study—eg numbers potentially eligible, examined for eligibility, confirmed eligible, included in the study, completing follow-up, and analysed	9
		(b) Give reasons for non-participation at each stage	9
		(c) Consider use of a flow diagram	N/A
Descriptive data	14*	(a) Give characteristics of study participants (eg demographic, clinical, social) and information on exposures and potential confounders	9
		(b) Indicate number of participants with missing data for each variable of interest	9, 17
Outcome data	15*	Report numbers of outcome events or summary measures	N/A
Main results	16	(*a*) Give unadjusted estimates and, if applicable, confounder-adjusted estimates and their precision (eg, 95% confidence interval). Make clear which confounders were adjusted for and why they were included	9-11, 18-19

For peer review only - http://bmjopen.bmj.com/site/about/guidelines.xhtml

		(*b*) Report category boundaries when continuous variables were categorized	
		(*c*) If relevant, consider translating estimates of relative risk into absolute risk for a meaningful time period	
Other analyses	17	Report other analyses done—eg analyses of subgroups and interactions, and sensitivity analyses	N/A
**Discussion**			
Key results	18	Summarise key results with reference to study objectives	11
Limitations	19	Discuss limitations of the study, taking into account sources of potential bias or imprecision. Discuss both direction and magnitude of any potential bias	12-13
Interpretation	20	Give a cautious overall interpretation of results considering objectives, limitations, multiplicity of analyses, results from similar studies, and other relevant evidence	13
Generalisability	21	Discuss the generalisability (external validity) of the study results	13
**Other information**			
Funding	22	Give the source of funding and the role of the funders for the present study and, if applicable, for the original study on which the present article is based	15

*Give information separately for exposed and unexposed groups.

Note: An Explanation and Elaboration article discusses each checklist item and gives methodological background and published examples of transparent reporting. The STROBE checklist is best used in conjunction with this article (freely available on the Web sites of PLoS Medicine at http://www.plosmedicine.org/, Annals of Internal Medicine at http://www.annals.org/, and Epidemiology at http://www.epidem.com/). Information on the STROBE Initiative is available at www.strobe-statement.org.

For peer review only

Stages to increase uptake of the schools-based Human Papillomavirus (HPV) vaccination programme: Are they successful and do they decrease inequalities in uptake?

Journal:	*BMJ Open*
Manuscript ID	bmjopen-2020-044980.R1
Article Type:	Original research
Date Submitted by the Author:	16-Mar-2021
Complete List of Authors:	Fisher, Harriet; University of Bristol, Bristol Medical School<br>Evans, Karen; Sirona Care and Health CIC<br>Reynolds, Rosy; University of Bristol, Bristol Medical School<br>Yates, Julie; Public Health England<br>Roderick, Marion; University Hospitals Bristol and Weston NHS Foundation Trust<br>Ferrie, Jo; Public Health England<br>Macleod, John; University of Bristol, School of Social and Community Medicine<br>Hickman, Matthew; University of Bristol, School of Social and Community Medicine<br>Audrey, Suzanne; University of Bristol, Schoolof Social and Community Medicine
**Primary Subject Heading**:	Public health
Secondary Subject Heading:	Immunology (including allergy)
Keywords:	PUBLIC HEALTH, INFECTIOUS DISEASES, Community child health < PAEDIATRICS, Public health < INFECTIOUS DISEASES

I, the Submitting Author has the right to grant and does grant on behalf of all authors of the Work (as defined in the below author licence), an exclusive licence and/or a non-exclusive licence for contributions from authors who are: i) UK Crown employees; ii) where BMJ has agreed a CC-BY licence shall apply, and/or iii) in accordance with the terms applicable for US Federal Government officers or employees acting as part of their official duties; on a worldwide, perpetual, irrevocable, royalty-free basis to BMJ Publishing Group Ltd ("BMJ") its licensees and where the relevant Journal is co-owned by BMJ to the co-owners of the Journal, to publish the Work in this journal and any other BMJ products and to exploit all rights, as set out in our licence.

The Submitting Author accepts and understands that any supply made under these terms is made by BMJ to the Submitting Author unless you are acting as an employee on behalf of your employer or a postgraduate student of an affiliated institution which is paying any applicable article publishing charge ("APC") for Open Access articles. Where the Submitting Author wishes to make the Work available on an Open Access basis (and intends to pay the relevant APC), the terms of reuse of such Open Access shall be governed by a Creative Commons licence – details of these licences and which Creative Commons licence will apply to this Work are set out in our licence referred to above.

Other than as permitted in any relevant BMJ Author's Self Archiving Policies, I confirm this Work has not been accepted for publication elsewhere, is not being considered for publication elsewhere and does not duplicate material already published. I confirm all authors consent to publication of this Work and authorise the granting of this licence.

For peer review only

TITLE PAGE

Title: Stages to increase uptake of the schools-based Human Papillomavirus (HPV) vaccination programme: Are they successful and do they decrease inequalities in uptake?

Dr Harriet Fisher[1]

Ms Karen Evans[2]

Dr Rosy Reynolds[1]

Dr Julie Yates[3]

Dr Marion Roderick[5]

Ms Jo Ferrie[3]

Professor John Macleod[1]

Professor Matthew Hickman[1]

Dr Suzanne Audrey[1]

[1] Population Health Sciences, Bristol Medical School, University of Bristol, Bristol, UK

[2] Head of School Nursing (Sirona Care & Health) and Specialist Nursing Services, Bristol and South Gloucestershire, UK

[3] Screening and Immunisations South West, Public Health England, Bristol, UK

[4] Bristol Biomedical Research Centre and NIHR CLAHRC West, Bristol, UK

[5] Department of Paediatric Immunology & Infectious Diseases, University Hospitals Bristol and Weston NHS Foundation Trust, Bristol, UK

Corresponding author:

Dr Harriet Fisher

Population Health Sciences, Bristol Medical School

University of Bristol

Canynge Hall

39 Whatley Road

For peer review only - http://bmjopen.bmj.com/site/about/guidelines.xhtml

Bristol, BS8 2PS

Email: Harriet.Fisher@bristol.ac.uk

Telephone: 0117 928 7223

Word count (main text): 3,576

Keywords: HPV vaccination programme; adolescents; consent; process evaluation; inequalities

For peer review only

ABSTRACT

Objectives: To test the impact on inequalities and uptake of the schools-based human papillomavirus (HPV) vaccination programme by stage of implementation of a new policy providing additional opportunities to consent.

Setting: Two local authorities in the south-west of England.

Participants: Young women (n=7,129) routinely eligible for HPV vaccination aged 12-13 years during the intervention period (2017/18 to 2018/19 programme years).

Interventions: Local policy change that included additional opportunities to provide consent (parental verbal consent and adolescent self-consent).

Outcomes: Secondary analyses of cross-sectional intervention data were undertaken to examine uptake by: (i) receipt of parental written consent forms, and; (ii) percentage of unvaccinated young women by stage of implementation.

Results: During the intervention period, 6,341 (89.0%) eligible young women initiated the HPV vaccination series. Parental written consent forms were less likely to be returned where young women attended alternative education provider settings ($p<0.001$), belonged to Non-White British ethnic groups ($p<0.01$) or more deprived quintiles ($p<0.001$). Implementation of parental verbal consent and adolescent self-consent reduced the percentage of unvaccinated young women from 21.3% to 16.5% (risk difference: 4.8%). The effect was greater for young women belonging to the most deprived compared to the least deprived quintile (risk difference: 7.4% vs. 2.3%, $p<0.001$), and for young women classified as Unknown ethnic category compared to White British young women (6.7% vs. 4.2%, $p<0.001$). No difference was found for Non-White British young women (5.4%, $p<0.21$).

Conclusions: Local policy change to consent procedures that allowed parents to consent verbally and adolescents to self-consent overcame some of the barriers to vaccination of young women belonging to families less likely to respond to paper-based methods of gaining consent and at greater risk of developing cervical cancer.

Word count: 278

Keywords: HPV vaccination programme; adolescents; consent; process evaluation; inequalities

Name of the registry: ISRCTN registry

Trial registration number: 49086105

For peer review only - http://bmjopen.bmj.com/site/about/guidelines.xhtml

Date of registration: 12/01/2018

URL of trial registry record: www.isrctn.com/ISRCTN49086105

ARTICLE SUMMARY

Strengths and limitations of the study

This study utilises routinely collected data to examine the impact of local policy changes on inequalities in uptake of the HPV vaccination programme.

The intervention period (programme years 2017/18 – 2018/19) was relatively short.

Missing data on ethnicity could change the direction or size of the corresponding Odds Ratios.

INTRODUCTION

The Human Papillomavirus (HPV) vaccine currently used in England protects against infection from high-risk HPV types 16 and 18 which cause cancers affecting the cervix, vulva, vagina, penis, anus, and oral cavity. The vaccine also protects against types 6 and 11 which cause 90% of genital warts. High coverage of the English HPV vaccination programme for young women aged 12 to 13 years has been achieved. Recent evidence highlights the potential for HPV vaccination programmes to substantially reduce the incidence of cervical cancer [1, 2]. Based on emerging evidence for cost-effectiveness, in 2019/20 the HPV vaccination programme was expanded to include young men aged 12 to 13 years.

Despite generally good coverage, without concerted efforts to address lower uptake amongst some populations, pre-existing disparities in the incidence of cervical cancer by ethnicity and deprivation may increase [3-5]. We previously identified lower uptake by area and amongst some population groups, including minority ethnic groups [6]. Our research in schools with lower uptake showed complex socio-cultural factors can influence whether young women are vaccinated [7] and the requirement for written parental consent may act as a barrier to some young women receiving the HPV vaccine [8].

In the United Kingdom (UK) (comprising England, Scotland, Northern Ireland, and Wales), consent for schools-based adolescent vaccination programmes is usually obtained from parents or carers. Young

For peer review only - http://bmjopen.bmj.com/site/about/guidelines.xhtml

people are provided with a form for their parent to sign and hand back to school before a vaccination session takes place. However, the UK legal framework allows young people to be vaccinated without parental consent provided they are deemed 'Gillick competent' [9] (i.e. they have sufficient maturity and intelligence to understand the nature and implications of the treatment). Findings from an evidence synthesis showed the implementation of adolescent self-consent procedures could be prevented by local policies which favoured written parental consent, and the precedence given to professionals' concerns about their reputations and relationships with parents [10].

A local authority is an organisation officially responsible for all public services and facilities in a particular area. To address concerns about lower uptake in two local authority areas in the south-west of England, a new policy (including parental verbal consent and adolescent self-consent) has been implemented [9]. Implementation of the new consent procedures in one of the intervention areas appeared to improve uptake in contrast to trends of decreasing uptake among matched local authorities [11]. However, no evidence for an absolute increase, or reduction in inequalities by deprivation and ethnicity was found.

The aim of the current study is to further examine the impact of the consent procedures on HPV vaccine uptake. Specifically, we describe:

(i) Receipt of parental written consent forms by school category, ethnicity and deprivation quintile

(ii) Unvaccinated young women by stage of implementation of consent procedures, and by school category, ethnicity and deprivation quintile

METHODS

This cross-sectional study was undertaken when the English vaccination programme was delivered routinely to young women only. Details of the evaluation and changes to the new local policy are provided in a published protocol [12].

The new local policy (including consent procedures)

In brief, previously only young women who had returned a written parental consent form, indicating the parent or carer is willing for their daughter to receive the HPV vaccine, were administered the vaccine in the school setting. Under the new arrangements, all young women eligible for the vaccination, including those whose parents have provided written refusal, are asked to attend the session by the immunisation team. For those young women who do not have a returned parental written consent form, the immunisation nurse attempts to gain parental verbal consent over the telephone. If the parent cannot be contacted and the young woman expresses willingness to be vaccinated, the immunisation nurse assesses the young woman's competence and if they are deemed competent the young women will receive the vaccine. All young women who do not receive the vaccine on the day, are given information about alternative options to receive the vaccination, such as through their family doctor or community-based clinics run by the immunisation team (Supplementary material 1). Parents are not routinely contacted again by the immunisation team.

We define stage of implementation of consent procedures as the following: (i) 'Stage One: parent written consent only'; (ii) 'Stage Two: parent verbal consent & adolescent self-consent', and; (iii) 'Stage Three: community catch up clinics and family practice settings'. These stages represent sequential opportunities (in a single programme) for unvaccinated young women to receive the vaccine, rather than different time-points.

Patient and public involvement

The Bristol Young Person's Advisory Group (YPAG) (website: https://generationr.org.uk/bristol/) took part in preliminary discussions about self-consent procedures, at which a group of 11 young people discussed self-consent for vaccination of young people who are of secondary school age. Following this discussion, all voted in favour of self-consent preferably with parents being informed. The young people were overall positive about the documents which they thought provided the right amount of information clearly. As a result of the feedback, some changes to the wording of the information and formatting were made. Members of the Bristol Youth Council (website: https://www.bristol.gov.uk/youth-council-youth-mayors) were approached to participate in the study during recruitment. This resulted in an opportunity to obtain feedback on a proposal developed from the findings of the current study.

For peer review only - http://bmjopen.bmj.com/site/about/guidelines.xhtml

During the study, the lead researcher was also invited to deliver school-based information sessions about the HPV vaccine to Year 7 students. The study researcher also led PHSE lessons where Year 8 students were able to find out about research and encouraged to debate issues around adolescent self-consent. Study findings are being shared with the YPAG and the Bristol City Youth Council at meetings to mark the end of the study.

Population

Two local authorities implementing the new consent procedures for the HPV vaccination programme in the south-west of England provided data. Records relating to young women eligible (born between 1st September 2004 and 31st August 2006) for vaccination during programme years 2017/18 and 2018/19 and who were registered with a general practice within the local authority boundaries were retrieved in July 2019 from the Child Health Information System.

Data extraction from the Child Health Information System

Prior to study commencement, permission to access an anonymised data extract was gained from the relevant organisations with responsibility for the data. In the UK, the Child Health Information System holds demographic and vaccination-related records for each young person registered with a family doctor which is a statutory requirement. The following data fields were extracted from records of the eligible population: (i) partial date of birth; (ii) partial postcode; (iii) ethnicity; (iv) dates and location HPV vaccination administered, and; (v) name and corresponding identifying code of school.

School identifying codes were used to assign the local authority responsible for delivery of the HPV vaccine. Partial date of birth was used to allocate programme year the young woman was eligible to receive the HPV vaccine. Categories of school types were applied to each record: (i) comprehensive, non-fee-paying; (ii) private, fee-paying, and; (iii) alternative education provider, which included pupil referral units, young offender units, hospital education service, specialist schools for students with significant additional needs and young women educated at home.

Individual records were classed as 'received HPV vaccine' if there was a record of at least one dose administered within the corresponding programme year the young woman was eligible. Postcodes from

For peer review only - http://bmjopen.bmj.com/site/about/guidelines.xhtml

individual records were linked to the corresponding Lower Super Output Area (LSOA). Deprivation score was assigned using the Index of Multiple Deprivation 2019 (a statistic on relative deprivation in small areas of England) [13] and analysed as quintiles. Due to small numbers, ethnicity was grouped as follows: (i) White British; (ii) Non-White British, and; (iii) Unknown.

Records were excluded if the relevant school identifying code was missing or invalid. Absence of recorded ethnicity was considered likely Missing Not At Random as absence of ethnicity data was associated with the outcome, school and deprivation variables. A complete case approach where records were excluded on the basis of missing ethnicity is not recommended [14]. Instead, the 'Unknown' category was assigned to missing ethnicity data to minimise the risk of bias.

Data extraction from the immunisation team's records of consent

Additional data was sought from the school immunisation team's electronic and paper-based records relating to vaccination consent during the intervention period. This included: (i) return of parental consent forms ('yes' or 'no') and; (ii) stage of implementation of consent procedures. We classified each record as belonging to one of the following stages of the consent procedure: (i) 'Stage One: parent written consent only'; (ii) 'Stage Two: parent verbal consent & adolescent self-consent', and; (iii) 'Stage Three: community catch up clinics and family practice settings'. Records could not be assigned to more than one stage of consent category.

Data linkage

The data extracted from the immunisation team's records were linked to the Child Health Information System using deterministic data linkage methods by a member of staff at Health Intelligence. An anonymised version of the data extract was securely transferred to researchers at the University of Bristol.

ANALYSIS

Return of parental consent forms

Logistic univariable analyses and likelihood ratio tests were performed to explore factors associated with return of parental consent form. The following explanatory variables for analysis were selected *a*

For peer review only - http://bmjopen.bmj.com/site/about/guidelines.xhtml

priori: school category, ethnicity and deprivation quintile. A multivariable logistic regression model was developed. We used cluster-robust errors in the final model to allow for the possibility of clustering within schools.

Unvaccinated young women by stage of implementation of consent procedures

To describe the decrease in unvaccinated young women at each stage of implementation of the consent procedures, we calculated risk differences (difference in two proportions) with 95% confidence intervals. The risk difference shows the absolute effect of implementation of each stage of the consent procedure. We considered the following risk differences (risk reductions) by: (i) percentage of young women unvaccinated during '*Stage One: parent written consent only*' minus percentage of young women unvaccinated during '*Stage One' and 'Stage Two: parent verbal consent & adolescent self-consent*', and; (ii) percentage of young women unvaccinated during '*Stage One'* minus percentage of young women unvaccinated during *'Stage One', 'Stage Two' and 'Stage Three: community catch up clinics and family practices'*).

To show whether there was an unintended increase or reduction in health inequalities, we compared the risk differences and corresponding p-values by school category, ethnic group, and deprivation quintile – comparing with a baseline category in each case.

Analyses were undertaken using the Stata statistical package, release 15 (Stata Corp, College Station, TX).

RESULTS

Data were extracted relating to 7,549 young women eligible for vaccination during the intervention period (programme years 2017/18 to 2018/19). Of these, 420 (5.6%) were excluded on the basis that the school data was missing or invalid.

Of the cohort retained for analysis (n=7,129), the majority of vaccine eligible young women were resident in local authority one (4,516, 63.4%), attended comprehensive, non-fee paying schools (6,350, 89.1%), and were classified as belonging to a White British ethnic group (4,888, 68.6%). Of young

For peer review only - http://bmjopen.bmj.com/site/about/guidelines.xhtml

women eligible for vaccination, 6,341 (89.0%) were recorded to have received the HPV vaccine during the programme year they were eligible. Parental consent forms were recorded as being unreturned (comprising active refusal and passive non-consent) for 1,555 (16.2%) of eligible young women (Table 1).

Return of parental consent forms

Variables associated with return of parental consent forms are provided as unadjusted Odds Ratios in Table 2. After adjusting for school category, ethnicity, and deprivation, an association was found between parental consent form not being returned and: attending an alternative education provider setting (adjusted Odds Ratio (aOR): 5.54, 95% CI: 3.80-8.09, p<0.001), belonging to a Non-White British (aOR: 1.34, 95% CI: 1.06-1.70, p<0.01), and Unknown ethnicity category (aOR: 2.41, 95% CI: 2.09-2.78, p<0.001). There was also evidence for a relationship with level of deprivation. For example, young women belonging to the most deprived quintile had at least double the odds of having a record of unreturned consent form (aOR: 2.54, 95% CI: 2.03-3.18, p<0.001) compared to those from the least deprived quintile (Table 2).

Percentage of young women unvaccinated by stage of implementation of consent procedures

Following implementation of Stage One (parental written consent) of the consent procedures, 1,519 (21.3%) of young women were unvaccinated. At Stage Two (parental verbal consent and adolescent self-consent), this reduced to 1,173 (16.5%) unvaccinated young women. With the inclusion of Stage Three (community catch up clinics and family practice settings), there remained 788 (11.1%) unvaccinated young women during the study period (Table 3). Not all parents could be contacted by the immunisation team on the day of the vaccination session (n=362). These parents were not routinely contacted again by the immunisation team (data not shown).

The percentage of unvaccinated young women varied by school category at different stages of implementation of the consent procedures. For example, at Stage One (parental written consent only), 20.6% of young women who attended mainstream comprehensive, non-fee-paying schools were unvaccinated, in comparison to 50.8% who attended alternative education provider settings. At Stage Two (parental verbal consent and adolescent self-consent), there was no evidence for narrowing of this

For peer review only - http://bmjopen.bmj.com/site/about/guidelines.xhtml

gap (p=0.27). However, after Stage Three (community catch-up clinics and family practice settings), the decrease in unvaccinated young women was greater for those that attended alternative education provider settings, compared to those who attended comprehensive, non-fee paying schools (accumulative risk difference: 23.7% vs. 10.2%, p<0.001) (Figure 1 & Table 3).

There were also differences by ethnicity. At Stage One (parental written consent only), 15.8% of White British young women were unvaccinated, in comparison to 26.7% of Non-White British young women and 35.7% belonging to the Unknown ethnic category. Following implementation of Stage Two (parental verbal consent and adolescent self-consent), the percentage unvaccinated decreased at a greater rate in young women classified as 'Unknown' ethnic category compared to White British young women (risk difference: 6.7% vs 4.2%, p<0.001), but no difference was observed for Non-White British young women (p=0.21). There was evidence for a difference with the inclusion of Stage Three for Non-White British women (accumulative risk difference: 12.8% vs. 8.9%, p=0.01) and Unknown ethnicity (accumulative risk difference: 13.4% vs. 8.9%, p<0.001) (Figure 1 & Table 3).

Inequalities in the percentage of unvaccinated young women by deprivation were attenuated by each stage of implementation of the policy. At Stage One (parental written consent only), 14.2% young women in the least deprived quintile were unvaccinated in comparison to 29.8% of young women in the most deprived quintile. Subsequent to Stage Two (parental verbal consent and adolescent self-consent), the percentage of unvaccinated young women decreased at a greater rate for those belonging to the most deprived quintile compared to the least deprived quintile (risk difference: 7.4% vs. 2.3%, p<0.001). A similar pattern was observed following implementation of Stage Three (community catch-up clinics and family practice settings) (overall risk difference: 13.8% vs. 5.8%, p<0.001) (Figure 1 & Table 3).

DISCUSSION

The HPV vaccination programme has been implemented to prevent HPV, a recognised precursor to developing cervical cancer. The findings from this study show that some of the barriers to young women being vaccinated were overcome through the implementation of a new local policy, which included parental verbal consent and adolescent self-consent in the school setting. There is promising evidence that the additional steps have the potential to reduce existing inequalities in uptake among young

For peer review only - http://bmjopen.bmj.com/site/about/guidelines.xhtml

women living in more deprived areas. Importantly, this study showed that these young women are less likely to engage with consent procedures that rely on paper-based methods, are more likely to be affected by cervical cancer [15, 16], and less likely to receive the HPV vaccine [6].

The majority of young women were vaccinated in the school setting. However, this study supports the provision of the HPV vaccine in community settings, such as catch-up clinics and general practice surgeries, to help improve access to vaccination. This may benefit young women who may have had anxieties about being vaccinated without a parent present, or did not attend school on the day of the vaccination session.

Community provision of the HPV vaccine also appeared to reduce substantial inequalities in uptake among young women educated in alternative education provider settings. The reasons for this are unclear, but could relate to their lower school attendance, complex behavioural and physical health needs, or perceptions of safety of vaccination in the school setting due to interactions with other medical treatment. Although they comprise a small proportion of the overall vaccine-eligible population, they are a vulnerable population with substantially lower uptake and greater health inequalities which requires addressing.

As the majority of parents who could be contacted provided verbal consent, the data imply that the absence of a signed parental consent form cannot be assumed to mean the parent does not want their daughter to have the vaccine. Not all parents could be contacted by the immunisation team on the day of the vaccination session. Provision of additional resources to contact families ahead of the vaccination session could help reduce the proportion of families who are not contacted and help ensure that their daughter receives the HPV vaccine if they wish.

Relative to parental verbal consent, adolescent self-consent occurred infrequently. Our analyses of qualitative data from this study showed a strong presumption that parents should make decisions affecting the health of their children. The preferred age at which the HPV vaccination is administered (12-13 years) also contributed to reluctance in endorsing self-consent which was thought to have the potential to break down trust between parents and school staff, and within families [17]. This suggests

For peer review only - http://bmjopen.bmj.com/site/about/guidelines.xhtml

For peer review only

that unresolved issues could act as a barrier to widespread implementation of adolescent self-consent in other settings.

Our primary statistical analyses for this study showed that the new consent procedures increased uptake by 11% in one of the intervention local authorities, and appeared to overcome trends for decreasing uptake in matched sites [11]. Our secondary analyses of the process evaluation data reported here showed an additional 347 young women (4.8%) received the HPV vaccine in the school setting. All things being equal, if changes in policy resulted in similar effect sizes in other local authorities, as part of a strategy to increase uptake, then the English HPV vaccination programme could reach the World Health Organisation's target of 90% of young women receiving the vaccination by 15 years old [18].

Additionally, establishing the cost-effectiveness of strategies to improve uptake of vaccination programmes is important to provide evidence for policy makers to target resources appropriately. This has been established in the context of the United States of America [19], but these findings are not easily translatable to English schools-based, rather than healthcare-based, vaccination programmes.

Public Health England have recently issued updated guidance for healthcare professionals related to the new universal HPV vaccination programme [20]. This supports the use of parent verbal consent and adolescent self-consent as strategies to maximise uptake and reduce catch-up sessions. They cite further benefit of inclusion of young people whose parents may have difficulties in completing the consent due to language or literacy issues. The findings from this study provide evidence that strategies incorporating parent verbal consent could help young women belonging to 'harder-to-reach' families receive the HPV vaccine. These recommendations may also be applicable to other schools-based vaccination programmes, including the influenza vaccination programme offered to primary school aged children where similar patterns in forms returns have been reported [21].

Strengths and limitations

The study has some strengths. This is the first study to examine how new local polices for the HPV vaccination programme are implemented, and the impact on health inequalities among more deprived

For peer review only - http://bmjopen.bmj.com/site/about/guidelines.xhtml

populations and young women belonging to minority ethnicity groups. Our study utilised routinely collected data related to vaccination status, eliminating the risk of recall and selection bias. The data relate to vaccinations delivered in school and community settings to all young women eligible for routine HPV vaccination during the study period. As such, our results correspond to an almost complete population.

There are some limitations. The data related to implementation of new consent procedures in a geographically distinct area in the south-west of England. The findings therefore may not be applicable to local authorities that are implementing new consent procedures in schools-based vaccination programmes elsewhere in the United Kingdom. The findings may also not translate to other adolescent vaccination programmes delivered in countries where cultural differences may influence the acceptability of parental verbal consent and adolescent self-consent procedures in the school setting.

As the study relied on routinely collected information, we did not have access to individual-level measures of socioeconomic status and relied on area-based measures of deprivation. Our study findings may therefore be subject to ecological fallacy.

An issue, common to all routinely collected data, is the possibility of data input errors and missing data. To minimise bias from inclusion of this data, we excluded almost 5% of the data as the information related to school was out-of-date. Overall, our dataset identified 8% of young women belonging to a minority ethnic group. This compares with nationally reported figures indicating 30% of young people attending secondary schools in the intervention areas belong to a non-White ethnic group [22]. Missing ethnicity data (23%) relating to young women who were born outside the local authority boundaries could change the direction or size of aORs corresponding to ethnicity.

Conclusions

Introducing further steps to the consent procedures – allowing parents to consent verbally and adolescent self-consent – overcame some of the barriers to vaccination of young women belonging to families less likely to respond to paper-based methods of gaining consent.

For peer review only - http://bmjopen.bmj.com/site/about/guidelines.xhtml

REFERENCES

1. Drolet, M., et al., Population-level impact and herd effects following the introduction of human papillomavirus vaccination programmes: updated systematic review and meta-analysis. The Lancet, 2019. **394**(10197): p. 497-509.

2. Simms, K.T., et al., Impact of scaled up human papillomavirus vaccination and cervical screening and the potential for global elimination of cervical cancer in 181 countries, 2020;99: a modelling study. The Lancet Oncology, 2019. **20**(3): p. 394-407.

3. Johnson, H.C., et al., Effect of HPV vaccination and cervical cancer screening in England by ethnicity: a modelling study. The Lancet Public Health, 2018. **3**(1): p. e44-e51.

4. Baker, D. and E. Middleton, Cervical screening and health inequality in England in the 1990s. Journal of Epidemiology and Community Health 2003. **57**(6): p. 417-423.

5. Shack, L., et al., Variation in incidence of breast, lung and cervical cancer and malignant melanoma of skin by socioeconomic group in England. BMC Cancer, 2008. **8**(1): p. 271.

6. Fisher, H., et al., Examining inequalities in the uptake of the school-based HPV vaccination programme in England: a retrospective cohort study. Journal of Public Health, 2013. **36**(1): p. 36-45.

7. Batista Ferrer, H., et al., Barriers and facilitators to uptake of the school-based HPV vaccination programme in an ethnically diverse group of young women. Journal of Public Health, 2016. **38**(3): p. 569-577.

8. Batista Ferrer, H., Factors influencing the uptake of the Human Papillomavirus (HPV) vaccination programme. Thesis (Ph.D.), 2014.

9. Gillick V . West Norfolk and Wisbech Area Health Authority and Department of Health and Social Security. 1985, House of Lords: London.

10. Fisher, H., et al., Barriers and enablers to adolescent self-consent for vaccination: A mixed-methods evidence synthesis. Vaccine, 2019. **37**(3): p. 417-429.

11. Fisher, H., et al., Impact of new consent procedures on uptake of the schools-based human papillomavirus (HPV) vaccination programme. Journal of Public Health, 2020.

12. Audrey, S., et al., Impact and acceptability of self-consent procedures for the school-based human papillomavirus vaccine: a mixed-methods study protocol. BMJ Open. 2018. **8**(3): p. e021321.

13. English indices of deprivation, 2019. Available from: https://www.gov.uk/government/statistics/english-indices-of-deprivation-2019.

14. Hughes, R.A., et al., Accounting for missing data in statistical analyses: multiple imputation is not always the answer. International Journal of Epidemiology, 2019. **48**(4): p. 1294-1304.

15. Shack, L., et al., Variation in incidence of breast, lung and cervical cancer and malignant melanoma of skin by socioeconomic group in England. BMC Cancer, 2008. **8**: p. 271.

16. Cancer Research UK and National Cancer Intelligence Network. Cancer by deprivation in England: Incidence, 1996-2010, Mortality, 1997-2011. 2014, NCIN: London.

17. Audrey, S., et al., How acceptable is adolescent self-consent for the HPV vaccination: qualitative findings from a process evaluation in south-west England. Vaccine, 2020. https://doi.org/10.1016/j.vaccine.2020.09.074.

18. World Health Organisation, Strategic Advisory Group of Experts on Immunization: Working Group on Human Papillomavirus Immunization, 2018. Available from:

For peer review only - http://bmjopen.bmj.com/site/about/guidelines.xhtml

https://www.who.int/immunization/sage/meetings/2018/october/3_SAGE2018_WG_recommendation_FINAL.pdf.

19. Spencer, J.C., et al., Cost-effectiveness of Interventions to Increase HPV Vaccine Uptake. Pediatrics. 2020. **146**(6): p. e20200395.

20. Public Health England, HPV vaccination: Information for healthcare practitioners. 2019, Available from: https://assets.publishing.service.gov.uk/government/uploads/system/uploads/attachment_data/file/813014/PHE_HPV_universal_programme_guidance.pdf.

21. Christensen, H., et al., Influence of commissioned provider type and deprivation score on uptake of the childhood flu immunization. Journal of Public Health, 2019.

22. Department for Education, Schools, pupils and their characteristics: January 2018. Avaialble from: https://www.gov.uk/government/statistics/schools-pupils-and-their-characteristics-january-2018.

DECLARATIONS

Consent & ethical considerations

Research Ethics Committee approval is not required for evaluations that are designed solely to define or judge current care and measure a current service without reference to a standard. This evaluation involves the retrospective analysis of an anonymised dataset comprising data routinely collated by the immunisation teams and the Child Health Information Services as part of the HPV vaccination programme. The study did not involve the collection of additional data. Research Ethics Committee approval was therefore not required. However, permission for the research team at the University of Bristol to receive an anonymised dataset was sought from the organisations assigned as data custodians for the data (Sirona Health and Care and In Health Intelligence).

Patient consent

Not required.

Data sharing statement

As the analysis was undertaken on routine data acquired from an external source, the study authors assured the data custodians that the dataset would be treated as confidential and would not be shared.

Competing interests

The authors have no competing interests to declare.

For peer review only - http://bmjopen.bmj.com/site/about/guidelines.xhtml

Funding

This work is funded by the National Institute for Health Research's Research for Patient Benefit (NIHR RfPB) programme (project number PB-PG-0416-20013). The views and opinions expressed therein are those of the authors and do not necessarily reflect those of the NIHR RfPB Programme or the Department of Health.

Contributorship statement

SA, HF, MH, KE, JF & MR were involved in the conception and design of the research. SA is principal investigator; HF is study manager and undertook data cleaning, analysed the data, and drafted the manuscript; JF, JY and KE developed and advised on the new consent procedures and the HPV vaccination process; MR advised on local immunisation strategy; JM advised on health inequalities and MH and RR advised on statistical methods. All authors have made substantial contributions to interpreting the data, revising it for important intellectual content, and have given approval of the final version to be submitted.

Acknowledgements

The authors express their gratitude to staff at In Health Intelligence for data linkage and extracting the data from the Child Health Information System, and to the immunisation and administrative teams at Sirona Healthcare who facilitated access to the immunisation consent records as part of this study.

The study is partly supported by the NIHR Health Protection Research Unit in Evaluation of Interventions at University of Bristol. This research was supported by the National Institute for Health Research (NIHR) Applied Research Collaboration West (NIHR ARC West). The views expressed in this article are those of the author(s) and not necessarily those of the NIHR or the Department of Health and Social Care. The work was undertaken with the support of The Centre for the Development and Evaluation of Complex Interventions for Public Health Improvement (DECIPHer), a UKCRC Public Health Research Centre of Excellence. Joint funding (MR/KO232331/1) from the British Heart Foundation, Cancer Research UK, Economic and Social Research Council, Medical Research Council,

For peer review only - http://bmjopen.bmj.com/site/about/guidelines.xhtml

the Welsh Government and the Wellcome Trust, under the auspices of the UK Clinical Research Collaboration, is gratefully acknowledged.

For peer review only

For peer review only - http://bmjopen.bmj.com/site/about/guidelines.xhtml

Table 1. Descriptive summary of eligible cohort by vaccine receipt and return of parent consent form

	**Eligible cohort n (%)**	**HPV vaccine received n (%)**	**Unreturned parent consent form n (%)**
	7,129 (100.0)	6,341 (89.0)	1,155 (16.2)
Area-level			
Local Authority One	4,516 (63.4)	3,944 (87.3)	843 (18.7)
Local Authority Two	2,613 (36.7)	2,397 (91.7)	312 (11.9)
Programme year 2017/18	3,581 (50.2)	3,202 (89.4)	565 (15.8)
Programme year 2018/19	3,548 (49.8)	3,139 (88.5)	590 (16.6)
School category			
Comprehensive, non-fee-paying	6,350 (89.1)	5,690 (89.6)	992 (15.6)
Private, fee-paying	661 (9.3)	565 (85.5)	105 (15.9)
Alternative education providers	118 (1.7)	86 (72.9)	58 (49.2)
Individual-level			
Ethnicity			
White British	4,888 (68.6)	4,552 (93.1)	610 (12.5)
Non-White British	572 (8.0)	492 (86.0)	101 (17.7)
Unknown	1,669 (23.4)	1,297 (77.7)	444 (26.6)
Deprivation			
Least deprived	1,348 (18.9)	1,235 (91.6)	136 (10.1)
Quintile 2	1,379 (19.3)	1,277 (92.6)	149 (10.8)
Quintile 3	1,403 (19.7)	1,273 (90.7)	210 (15.0)
Quintile 4	1,396 (19.6)	1,203 (86.2)	292 (20.9)
Most deprived	1,421 (19.9)	1,194 (84.0)	338 (23.8)
Unknown	182 (3.6)	159 (87.4)	30 (16.5)

For peer review only

For peer review only - http://bmjopen.bmj.com/site/about/guidelines.xhtml

Table 2. Associations of unreturned parental consent form with school category, ethnicity & deprivation

	N (%)	Form not returned	OR (95% CI) †	p-value	aOR (95% CI) †	p-value
			-	-	-	-
School category		**n (%)**				
Comprehensive, non-fee-paying	6,350	992 (15.6)	-	-		
Private, fee-paying	661	105 (15.9)	1.02 (0.82-1.27)	0.86	1.12 (0.89-1.43)	0.34
Alternative education providers	118	58 (49.2)	5.22 (3.62-7.54)	<0.001	5.54 (3.80-8.09)	<0.001
Ethnicity						
White British	4,888	610 (12.5)	-	-	-	-
Non-White British	572	101 (17.7)	1.50 (1.19-1.89)	<0.01	1.34 (1.06-1.70)	0.01
Unknown	1,669	444 (26.6)	2.54 (2.21-2.92)	<0.001	2.41 (2.09-2.78)	<0.001
Deprivation						
Least deprived	1,279	136 (10.1)	-		-	
Quintile 2	1,324	149 (10.8)	1.08 (0.84-1.38)	0.54	1.09 (0.85-1.40)	0.48
Quintile 3	1,334	210 (15.0)	1.57 (1.25-1.97)	<0.001	1.57 (1.24-1.98)	<0.001
Quintile 4	1,305	292 (20.9)	2.36 (1.89-2.93)	<0.001	2.24 (1.79-2.81)	<0.001
Most deprived	1,347	338 (23.8)	2.78 (2.24-3.45)	<0.001	2.54 (2.03-3.18)	<0.001
Unknown	182	30 (16.4)	1.75 (1.14-2.70)	0.01	1.52 (0.97-2.37)	0.07

† Adjusted for ethnicity, deprivation, school category, and clustering by school; OR: Odds Ratio; CI: Confidence Intervals; aOR: Adjusted Odds Ratio;

For peer review only

For peer review only - http://bmjopen.bmj.com/site/about/guidelines.xhtml

Table 3. Percentage of young women unvaccinated by implementation stage of consent process

		**Stage 1†**	**Stages 1 & 2**	**Risk reduction[1]**	**p-value[1]**	**Stages 1, 2 & 3**	**Risk reduction[2]**	**p-value[2]**
		**n (%)**	**n (%)**	**n (%)**		**n (%)**	**n (%)**	**-**
		1,519 (21.3)	1,173 (16.5)	347 (4.8)		788 (11.1)	731 (10.3)	
School category	**N (%)**	**% (95% CI)**	**% (95% CI)**	**% (95% CI)**	**-**	**% (95% CI)**	**% (95% CI)**	
Comprehensive, non-fee-paying	6,350	20.6 (19.6-21.6)	15.4 (14.5-16.3)	5.3 (4.7-5.8)	-	10.4 (9.7-11.2)	10.2 (9.5-11.0)	-
Private, fee-paying	661	22.7 (19.7-26.0)	21.5 (18.5-24.8)	1.2 (0.6-2.4)	<0.001	14.5 (12.0-17.4)	8.2 (6.3-10.5)	0.07
Alternative education providers	118	50.8 (41.9-59.7)	47.5 (38.7-56.4)	3.4 (1.3-8.7)	0.27	27.1 (19.9-35.8)	23.7 (16.9-32.3)	<0.001
Ethnicity								
White British	4,705	15.8 (14.8-16.8)	11.6 (10.7-12.5)	4.2 (3.6-4.8)	-	6.9 (6.2-7.6)	8.9 (8.1-9.7)	-
Non-White British	542	26.7 (23.3-30.5)	21.3 (18.2-24.9)	5.4 (3.8-7.6)	0.21	14.0 (11.4-17.1)	12.8 (10.3-15.8)	0.01
Unknown	1,503	35.7 (33.4-38.0)	29.0 (26.9-31.2)	6.7 (5.6-8.0)	<0.001	22.3 (20.4-24.3)	13.4 (11.8-15.1)	<0.001
Deprivation								
Least deprived	1,279	14.2 (12.4-16.1)	11.9 (10.3-13.7)	2.3 (1.6-3.3)	-	8.4 (7.0-10.0)	5.8 (4.7-7.2)	-
Quintile 2	1,324	14.9 (13.1-16.8)	11.2 (9.7-13.0)	3.6 (2.8-4.8)	0.04	7.4 (6.1-8.9)	7.5 (6.2-9.0)	0.08
Quintile 3	1,334	19.8 (17.8-22.0)	14.9 (13.1-16.9)	4.9 (3.9-6.2)	<0.001	9.3 (7.9-10.9)	10.5 (9.0-12.3)	<0.001
Quintile 4	1,305	27.1 (24.8-29.5)	20.8 (18.8-23.1)	6.2 (5.1-7.6)	<0.001	13.8 (12.1-15.7)	13.3 (11.6-15.1)	<0.001
Most deprived	1,347	29.8 (27.4-32.2)	22.4 (20.3-24.6)	7.4 (6.1-8.9)	<0.001	16.0 (14.2-18.0)	13.8 (12.1-15.7)	<0.001
Unknown		24.2 (18.5-30.9)	22.0 (16.6-28.5)	12.6 (8.6-18.2)	0.93	12.6 (8.5-18.3)	11.5 (7.6-17.1)	0.02

† Stage 1: Parental written consent only; Stage 2: Schools-based procedures (parental verbal consent & adolescent self-consent; Stage 3: Community settings (catch-up clinics & family practices); CI: Confidence intervals;

[1]Risk difference for stages 1 & 2 compared to stage 1; p-value for comparison with risk difference in baseline group.

[2]Risk difference for stages 1 & 2 & 3 compared to stage 1; p-value for comparison with risk difference in baseline group.

For peer review only

Figure legends

Figure 1. Percentage of HPV vaccine eligible young women unvaccinated by stage of implementation of consent procedure

For peer review only

For peer review only - http://bmjopen.bmj.com/site/about/guidelines.xhtml

Figure 1. Percentage of HPV vaccine eligible young women unvaccinated by stage of implementation of consent procedure

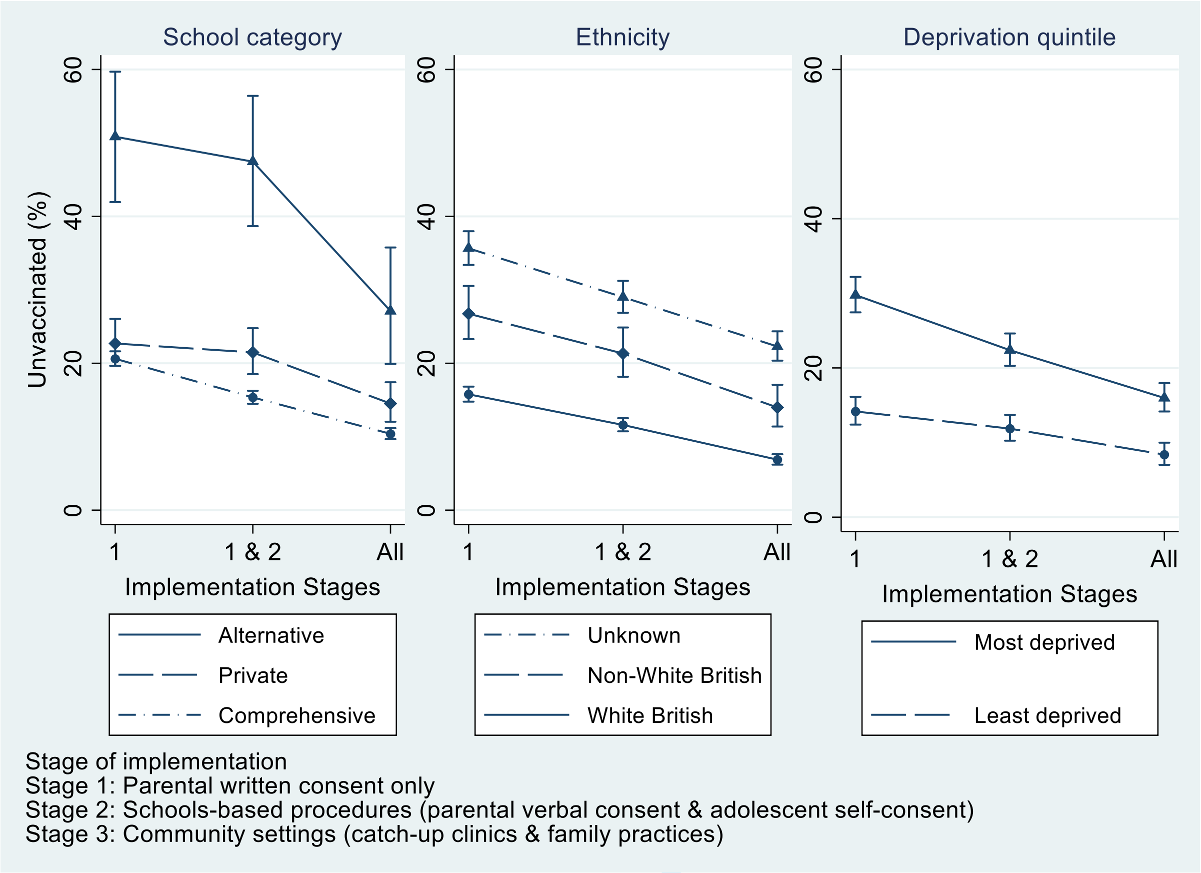

For peer review only

Supplementary material 1. Pathway of new consent procedures

Stage One

Parental written consent

- Consent forms sent home via school to parents
- School collate forms for the vaccination session
- Young women with signed consent forms receive the HPV vaccine

Stage Two

Parental verbal consent & adolescent self-consent

- For young women without signed consent forms, parents telephoned for opportunity to verbally consent
- Young women asked whether discussion about vaccination had taken place at home
- Young women assessed for self-consent by immunisation team

Stage Three

Community catch-up clinics & family practice

- Unvaccinated young women are provided with written information about community catch-up clinics
- Some young women may also choose to be vaccinated in the family practice setting

For peer review only

STROBE Statement—Checklist of items that should be included in reports of ***cross-sectional studies***

	Item No	Recommendation	Page No
**Title and abstract**	1	(*a*) Indicate the study's design with a commonly used term in the title or the abstract	3
		(*b*) Provide in the abstract an informative and balanced summary of what was done and what was found	3
**Introduction**			
Background/rationale	2	Explain the scientific background and rationale for the investigation being reported	4-5
Objectives	3	State specific objectives, including any prespecified hypotheses	5
**Methods**			
Study design	4	Present key elements of study design early in the paper	5
Setting	5	Describe the setting, locations, and relevant dates, including periods of recruitment, exposure, follow-up, and data collection	6-8
Participants	6	(*a*) Give the eligibility criteria, and the sources and methods of selection of participants	6-8
Variables	7	Clearly define all outcomes, exposures, predictors, potential confounders, and effect modifiers. Give diagnostic criteria, if applicable	6-8
Data sources/ measurement	8*	For each variable of interest, give sources of data and details of methods of assessment (measurement). Describe comparability of assessment methods if there is more than one group	6-8
Bias	9	Describe any efforts to address potential sources of bias	7
Study size	10	Explain how the study size was arrived at	7-9
Quantitative variables	11	Explain how quantitative variables were handled in the analyses. If applicable, describe which groupings were chosen and why	7-8
Statistical methods	12	(*a*) Describe all statistical methods, including those used to control for confounding	8-9
		(*b*) Describe any methods used to examine subgroups and interactions	8-9
		(*c*) Explain how missing data were addressed	7
		(*d*) If applicable, describe analytical methods taking account of sampling strategy	N/A
		(*e*) Describe any sensitivity analyses	N/A
**Results**			
Participants	13*	(a) Report numbers of individuals at each stage of study—eg numbers potentially eligible, examined for eligibility, confirmed eligible, included in the study, completing follow-up, and analysed	9
		(b) Give reasons for non-participation at each stage	9
		(c) Consider use of a flow diagram	N/A
Descriptive data	14*	(a) Give characteristics of study participants (eg demographic, clinical, social) and information on exposures and potential confounders	9
		(b) Indicate number of participants with missing data for each variable of interest	9, 17
Outcome data	15*	Report numbers of outcome events or summary measures	N/A
Main results	16	(*a*) Give unadjusted estimates and, if applicable, confounder-adjusted estimates and their precision (eg, 95% confidence interval). Make clear which confounders were adjusted for and why they were included	9-11, 18-19

For peer review only - http://bmjopen.bmj.com/site/about/guidelines.xhtml

For peer review only

		(*b*) Report category boundaries when continuous variables were categorized	
		(*c*) If relevant, consider translating estimates of relative risk into absolute risk for a meaningful time period	
Other analyses	17	Report other analyses done—eg analyses of subgroups and interactions, and sensitivity analyses	N/A
**Discussion**			
Key results	18	Summarise key results with reference to study objectives	11
Limitations	19	Discuss limitations of the study, taking into account sources of potential bias or imprecision. Discuss both direction and magnitude of any potential bias	12-13
Interpretation	20	Give a cautious overall interpretation of results considering objectives, limitations, multiplicity of analyses, results from similar studies, and other relevant evidence	13
Generalisability	21	Discuss the generalisability (external validity) of the study results	13
**Other information**			
Funding	22	Give the source of funding and the role of the funders for the present study and, if applicable, for the original study on which the present article is based	15

*Give information separately for exposed and unexposed groups.

Note: An Explanation and Elaboration article discusses each checklist item and gives methodological background and published examples of transparent reporting. The STROBE checklist is best used in conjunction with this article (freely available on the Web sites of PLoS Medicine at http://www.plosmedicine.org/, Annals of Internal Medicine at http://www.annals.org/, and Epidemiology at http://www.epidem.com/). Information on the STROBE Initiative is available at www.strobe-statement.org.

For peer review only

BMJ Open

Secondary analyses to test the impact on inequalities and uptake of the schools-based human papillomavirus (HPV) vaccination programme by stage of implementation of a new consent policy in the south-west of England

Journal:	*BMJ Open*
Manuscript ID	bmjopen-2020-044980.R2
Article Type:	Original research
Date Submitted by the Author:	15-Apr-2021
Complete List of Authors:	Fisher, Harriet; University of Bristol, Bristol Medical School<br>Evans, Karen; Sirona Care and Health CIC<br>Reynolds, Rosy; University of Bristol, Bristol Medical School<br>Yates, Julie; Public Health England<br>Roderick, Marion; University Hospitals Bristol and Weston NHS Foundation Trust<br>Ferrie, Jo; Public Health England<br>Macleod, John; University of Bristol, School of Social and Community Medicine<br>Hickman, Matthew; University of Bristol, School of Social and Community Medicine<br>Audrey, Suzanne; University of Bristol, Schoolof Social and Community Medicine
**Primary Subject Heading**:	Public health
Secondary Subject Heading:	Immunology (including allergy)
Keywords:	PUBLIC HEALTH, INFECTIOUS DISEASES, Community child health < PAEDIATRICS, Public health < INFECTIOUS DISEASES

SCHOLARONE™
Manuscripts

I, the Submitting Author has the right to grant and does grant on behalf of all authors of the Work (as defined in the below author licence), an exclusive licence and/or a non-exclusive licence for contributions from authors who are: i) UK Crown employees; ii) where BMJ has agreed a CC-BY licence shall apply, and/or iii) in accordance with the terms applicable for US Federal Government officers or employees acting as part of their official duties; on a worldwide, perpetual, irrevocable, royalty-free basis to BMJ Publishing Group Ltd ("BMJ") its licensees and where the relevant Journal is co-owned by BMJ to the co-owners of the Journal, to publish the Work in this journal and any other BMJ products and to exploit all rights, as set out in our licence.

The Submitting Author accepts and understands that any supply made under these terms is made by BMJ to the Submitting Author unless you are acting as an employee on behalf of your employer or a postgraduate student of an affiliated institution which is paying any applicable article publishing charge ("APC") for Open Access articles. Where the Submitting Author wishes to make the Work available on an Open Access basis (and intends to pay the relevant APC), the terms of reuse of such Open Access shall be governed by a Creative Commons licence – details of these licences and which Creative Commons licence will apply to this Work are set out in our licence referred to above.

Other than as permitted in any relevant BMJ Author's Self Archiving Policies, I confirm this Work has not been accepted for publication elsewhere, is not being considered for publication elsewhere and does not duplicate material already published. I confirm all authors consent to publication of this Work and authorise the granting of this licence.

For peer review only

TITLE PAGE

Title: Secondary analyses to test the impact on inequalities and uptake of the schools-based human papillomavirus (HPV) vaccination programme by stage of implementation of a new consent policy in the south-west of England

Dr Harriet Fisher[1]

Ms Karen Evans[2]

Dr Rosy Reynolds[1]

Dr Julie Yates[3]

Dr Marion Roderick[4]

Ms Jo Ferrie[3]

Professor John Macleod[1,5]

Professor Matthew Hickman[1]

Dr Suzanne Audrey[1]

[1] Population Health Sciences, Bristol Medical School, University of Bristol, Bristol, UK

[2] Head of School Nursing (Sirona Care & Health) and Specialist Nursing Services, Bristol and South Gloucestershire, UK

[3] Screening and Immunisations South West, Public Health England, Bristol, UK

[4] Department of Paediatric Immunology & Infectious Diseases, University Hospitals Bristol and Weston NHS Foundation Trust, Bristol, UK

[5] Bristol Biomedical Research Centre and NIHR CLAHRC West, Bristol, UK

Corresponding author:

Dr Harriet Fisher

Population Health Sciences, Bristol Medical School

University of Bristol

Canynge Hall

For peer review only

For peer review only - http://bmjopen.bmj.com/site/about/guidelines.xhtml

39 Whatley Road

Bristol, BS8 2PS

Email: Harriet.Fisher@bristol.ac.uk

Telephone: 0117 928 7223

Word count (main text): 3,576

Keywords: HPV vaccination programme; adolescents; consent; process evaluation; inequalities

For peer review only

ABSTRACT

Objectives: To test the impact on inequalities and uptake of the schools-based human papillomavirus (HPV) vaccination programme by stage of implementation of a new policy providing additional opportunities to consent.

Setting: Two local authorities in the south-west of England.

Participants: Young women (n=7,129) routinely eligible for HPV vaccination aged 12-13 years during the intervention period (2017/18 to 2018/19 programme years).

Interventions: Local policy change that included additional opportunities to provide consent (parental verbal consent and adolescent self-consent).

Outcomes: Secondary analyses of cross-sectional intervention data were undertaken to examine uptake by: (i) receipt of parental written consent forms, and; (ii) percentage of unvaccinated young women by stage of implementation.

Results: During the intervention period, 6,341 (89.0%) eligible young women initiated the HPV vaccination series. Parental written consent forms were less likely to be returned where young women attended alternative education provider settings ($p<0.001$), belonged to Non-White British ethnic groups ($p<0.01$) or more deprived quintiles ($p<0.001$). Implementation of parental verbal consent and adolescent self-consent reduced the percentage of unvaccinated young women from 21.3% to 16.5% (risk difference: 4.8%). The effect was greater for young women belonging to the most deprived compared to the least deprived quintile (risk difference: 7.4% vs. 2.3%, $p<0.001$), and for young women classified as Unknown ethnic category compared to White British young women (6.7% vs. 4.2%, $p<0.001$). No difference was found for Non-White British young women (5.4%, $p<0.21$).

Conclusions: Local policy change to consent procedures that allowed parents to consent verbally and adolescents to self-consent overcame some of the barriers to vaccination of young women belonging to families less likely to respond to paper-based methods of gaining consent and at greater risk of developing cervical cancer.

Word count: 278

Keywords: HPV vaccination programme; adolescents; consent; process evaluation; inequalities

Name of the registry: ISRCTN registry

Trial registration number: 49086105

For peer review only - http://bmjopen.bmj.com/site/about/guidelines.xhtml

Date of registration: 12/01/2018

URL of trial registry record: www.isrctn.com/ISRCTN49086105

ARTICLE SUMMARY

Strengths and limitations of the study

This study utilises routinely collected data to examine the impact of local policy changes on inequalities in uptake of the HPV vaccination programme.

The intervention period (programme years 2017/18 – 2018/19) was relatively short.

Missing data on ethnicity could change the direction or size of the corresponding Odds Ratios.

For peer review only

INTRODUCTION

The Human Papillomavirus (HPV) vaccine currently used in England protects against infection from high-risk HPV types 16 and 18 which cause cancers affecting the cervix, vulva, vagina, penis, anus, and oral cavity. The vaccine also protects against types 6 and 11 which cause 90% of genital warts. High coverage of the English HPV vaccination programme for young women aged 12 to 13 years has been achieved. Recent evidence highlights the potential for HPV vaccination programmes to substantially reduce the incidence of cervical cancer [1, 2]. Based on emerging evidence for cost-effectiveness, in 2019/20 the HPV vaccination programme was expanded to include young men aged 12 to 13 years.

Despite generally good coverage, without concerted efforts to address lower uptake amongst some populations, pre-existing disparities in the incidence of cervical cancer by ethnicity and deprivation may increase [3-5]. We previously identified lower uptake by area and amongst some population groups, including minority ethnic groups [6]. Our research in schools with lower uptake showed complex socio-cultural factors can influence whether young women are vaccinated [7] and the requirement for written parental consent may act as a barrier to some young women receiving the HPV vaccine [8].

In the United Kingdom (UK) (comprising England, Scotland, Northern Ireland, and Wales), consent for schools-based adolescent vaccination programmes is usually obtained from parents or carers. Young

For peer review only - http://bmjopen.bmj.com/site/about/guidelines.xhtml

people are provided with a form for their parent to sign and hand back to school before a vaccination session takes place. However, the UK legal framework allows young people to be vaccinated without parental consent provided they are deemed 'Gillick competent' [9] (i.e. they have sufficient maturity and intelligence to understand the nature and implications of the treatment). Findings from an evidence synthesis showed the implementation of adolescent self-consent procedures could be prevented by local policies which favoured written parental consent, and the precedence given to professionals' concerns about their reputations and relationships with parents [10].

A local authority is an organisation officially responsible for all public services and facilities in a particular area. To address concerns about lower uptake in two local authority areas in the south-west of England, a new policy (including parental verbal consent and adolescent self-consent) has been implemented [9]. Implementation of the new consent procedures in one of the intervention areas appeared to improve uptake in contrast to trends of decreasing uptake among matched local authorities [11]. However, no evidence for an absolute increase, or reduction in inequalities by deprivation and ethnicity was found.

The aim of the current study is to further examine the impact of the consent procedures on HPV vaccine uptake. Specifically, we describe:

(i) Receipt of parental written consent forms by school category, ethnicity and deprivation quintile

(ii) Unvaccinated young women by stage of implementation of consent procedures, and by school category, ethnicity and deprivation quintile

METHODS

This cross-sectional study was undertaken when the English vaccination programme was delivered routinely to young women only. Details of the evaluation and changes to the new local policy are provided in a published protocol [12].

The new local policy (including consent procedures)

For peer review only - http://bmjopen.bmj.com/site/about/guidelines.xhtml

In brief, previously only young women who had returned a written parental consent form, indicating the parent or carer is willing for their daughter to receive the HPV vaccine, were administered the vaccine in the school setting. Under the new arrangements, all young women eligible for the vaccination, including those whose parents have provided written refusal, are asked to attend the session by the immunisation team. For those young women who do not have a returned parental written consent form, the immunisation nurse attempts to gain parental verbal consent over the telephone. If the parent cannot be contacted and the young woman expresses willingness to be vaccinated, the immunisation nurse assesses the young woman's competence and if they are deemed competent the young women will receive the vaccine. All young women who do not receive the vaccine on the day, are given information about alternative options to receive the vaccination, such as through their family doctor or community-based clinics run by the immunisation team (Supplementary material 1). Parents are not routinely contacted again by the immunisation team.

We define stage of implementation of consent procedures as the following: (i) 'Stage One: parent written consent only'; (ii) 'Stage Two: parent verbal consent & adolescent self-consent', and; (iii) 'Stage Three: community catch up clinics and family practice settings'. These stages represent sequential opportunities (in a single programme) for unvaccinated young women to receive the vaccine, rather than different time-points.

Patient and public involvement

The Bristol Young Person's Advisory Group (YPAG) (website: https://generationr.org.uk/bristol/) took part in preliminary discussions about self-consent procedures, at which a group of 11 young people discussed self-consent for vaccination of young people who are of secondary school age. Following this discussion, all voted in favour of self-consent preferably with parents being informed. The young people were overall positive about the documents which they thought provided the right amount of information clearly. As a result of the feedback, some changes to the wording of the information and formatting were made. Members of the Bristol Youth Council (website: https://www.bristol.gov.uk/youth-council-youth-mayors) were approached to participate in the study during recruitment. This resulted in an opportunity to obtain feedback on a proposal developed from the findings of the current study.

only

For peer review only - http://bmjopen.bmj.com/site/about/guidelines.xhtml

During the study, the lead researcher was also invited to deliver school-based information sessions about the HPV vaccine to Year 7 students. The study researcher also led PHSE lessons where Year 8 students were able to find out about research and encouraged to debate issues around adolescent self-consent. Study findings are being shared with the YPAG and the Bristol City Youth Council at meetings to mark the end of the study.

Population

Two local authorities implementing the new consent procedures for the HPV vaccination programme in the south-west of England provided data. Records relating to young women eligible (born between 1st September 2004 and 31st August 2006) for vaccination during programme years 2017/18 and 2018/19 and who were registered with a general practice within the local authority boundaries were retrieved in July 2019 from the Child Health Information System.

Data extraction from the Child Health Information System

Prior to study commencement, permission to access an anonymised data extract was gained from the relevant organisations with responsibility for the data. In the UK, the Child Health Information System holds demographic and vaccination-related records for each young person registered with a family doctor which is a statutory requirement. The following data fields were extracted from records of the eligible population: (i) partial date of birth; (ii) partial postcode; (iii) ethnicity; (iv) dates and location HPV vaccination administered, and; (v) name and corresponding identifying code of school.

School identifying codes were used to assign the local authority responsible for delivery of the HPV vaccine. Partial date of birth was used to allocate programme year the young woman was eligible to receive the HPV vaccine. Categories of school types were applied to each record: (i) comprehensive, non-fee-paying; (ii) private, fee-paying, and; (iii) alternative education provider, which included pupil referral units, young offender units, hospital education service, specialist schools for students with significant additional needs and young women educated at home.

Individual records were classed as 'received HPV vaccine' if there was a record of at least one dose administered within the corresponding programme year the young woman was eligible. Postcodes from

For peer review only - http://bmjopen.bmj.com/site/about/guidelines.xhtml

individual records were linked to the corresponding Lower Super Output Area (LSOA). Deprivation score was assigned using the Index of Multiple Deprivation 2019 (a statistic on relative deprivation in small areas of England) [13] and analysed as quintiles. Due to small numbers, ethnicity was grouped as follows: (i) White British; (ii) Non-White British, and; (iii) Unknown.

Records were excluded if the relevant school identifying code was missing or invalid. Absence of recorded ethnicity was considered likely Missing Not At Random as absence of ethnicity data was associated with the outcome, school and deprivation variables. A complete case approach where records were excluded on the basis of missing ethnicity is not recommended [14]. Instead, the 'Unknown' category was assigned to missing ethnicity data to minimise the risk of bias.

Data extraction from the immunisation team's records of consent

Additional data was sought from the school immunisation team's electronic and paper-based records relating to vaccination consent during the intervention period. This included: (i) return of parental consent forms ('yes' or 'no') and; (ii) stage of implementation of consent procedures. We classified each record as belonging to one of the following stages of the consent procedure: (i) 'Stage One: parent written consent only'; (ii) 'Stage Two: parent verbal consent & adolescent self-consent', and; (iii) 'Stage Three: community catch up clinics and family practice settings'. Records could not be assigned to more than one stage of consent category.

Data linkage

The data extracted from the immunisation team's records were linked to the Child Health Information System using deterministic data linkage methods by a member of staff at Health Intelligence. An anonymised version of the data extract was securely transferred to researchers at the University of Bristol.

ANALYSIS

Return of parental consent forms

Logistic univariable analyses and likelihood ratio tests were performed to explore factors associated with return of parental consent form. The following explanatory variables for analysis were selected *a*

For peer review only - http://bmjopen.bmj.com/site/about/guidelines.xhtml

priori: school category, ethnicity and deprivation quintile. A multivariable logistic regression model was developed. We used cluster-robust errors in the final model to allow for the possibility of clustering within schools.

Unvaccinated young women by stage of implementation of consent procedures

To describe the decrease in unvaccinated young women at each stage of implementation of the consent procedures, we calculated risk differences (difference in two proportions) with 95% confidence intervals. The risk difference shows the absolute effect of implementation of each stage of the consent procedure. We considered the following risk differences (risk reductions) by: (i) percentage of young women unvaccinated during '*Stage One: parent written consent only*' minus percentage of young women unvaccinated during '*Stage One' and 'Stage Two: parent verbal consent & adolescent self-consent*', and; (ii) percentage of young women unvaccinated during '*Stage One'* minus percentage of young women unvaccinated during *'Stage One', 'Stage Two' and 'Stage Three: community catch up clinics and family practices'*).

To show whether there was an unintended increase or reduction in health inequalities, we compared the risk differences and corresponding p-values by school category, ethnic group, and deprivation quintile – comparing with a baseline category in each case.

Analyses were undertaken using the Stata statistical package, release 15 (Stata Corp, College Station, TX).

RESULTS

Data were extracted relating to 7,549 young women eligible for vaccination during the intervention period (programme years 2017/18 to 2018/19). Of these, 420 (5.6%) were excluded on the basis that the school data was missing or invalid.

Of the cohort retained for analysis (n=7,129), the majority of vaccine eligible young women were resident in local authority one (4,516, 63.4%), attended comprehensive, non-fee paying schools (6,350, 89.1%), and were classified as belonging to a White British ethnic group (4,888, 68.6%). Of young

For peer review only - http://bmjopen.bmj.com/site/about/guidelines.xhtml

women eligible for vaccination, 6,341 (89.0%) were recorded to have received the HPV vaccine during the programme year they were eligible. Parental consent forms were recorded as being unreturned (comprising active refusal and passive non-consent) for 1,555 (16.2%) of eligible young women (Table 1).

Return of parental consent forms

Variables associated with return of parental consent forms are provided as unadjusted Odds Ratios in Table 2. After adjusting for school category, ethnicity, and deprivation, an association was found between parental consent form not being returned and: attending an alternative education provider setting (adjusted Odds Ratio (aOR): 5.54, 95% CI: 3.80-8.09, p<0.001), belonging to a Non-White British (aOR: 1.34, 95% CI: 1.06-1.70, p<0.01), and Unknown ethnicity category (aOR: 2.41, 95% CI: 2.09-2.78, p<0.001). There was also evidence for a relationship with level of deprivation. For example, young women belonging to the most deprived quintile had at least double the odds of having a record of unreturned consent form (aOR: 2.54, 95% CI: 2.03-3.18, p<0.001) compared to those from the least deprived quintile (Table 2).

Percentage of young women unvaccinated by stage of implementation of consent procedures

Following implementation of Stage One (parental written consent) of the consent procedures, 1,519 (21.3%) of young women were unvaccinated. At Stage Two (parental verbal consent and adolescent self-consent), this reduced to 1,173 (16.5%) unvaccinated young women. With the inclusion of Stage Three (community catch up clinics and family practice settings), there remained 788 (11.1%) unvaccinated young women during the study period (Table 3). Not all parents could be contacted by the immunisation team on the day of the vaccination session (n=362). These parents were not routinely contacted again by the immunisation team (data not shown).

The percentage of unvaccinated young women varied by school category at different stages of implementation of the consent procedures. For example, at Stage One (parental written consent only), 20.6% of young women who attended mainstream comprehensive, non-fee-paying schools were unvaccinated, in comparison to 50.8% who attended alternative education provider settings. At Stage Two (parental verbal consent and adolescent self-consent), there was no evidence for narrowing of this

For peer review only - http://bmjopen.bmj.com/site/about/guidelines.xhtml

gap (p=0.27). However, after Stage Three (community catch-up clinics and family practice settings), the decrease in unvaccinated young women was greater for those that attended alternative education provider settings, compared to those who attended comprehensive, non-fee paying schools (accumulative risk difference: 23.7% vs. 10.2%, p<0.001) (Figure 1 & Table 3).

There were also differences by ethnicity. At Stage One (parental written consent only), 15.8% of White British young women were unvaccinated, in comparison to 26.7% of Non-White British young women and 35.7% belonging to the Unknown ethnic category. Following implementation of Stage Two (parental verbal consent and adolescent self-consent), the percentage unvaccinated decreased at a greater rate in young women classified as 'Unknown' ethnic category compared to White British young women (risk difference: 6.7% vs 4.2%, p<0.001), but no difference was observed for Non-White British young women (p=0.21). There was evidence for a difference with the inclusion of Stage Three for Non-White British women (accumulative risk difference: 12.8% vs. 8.9%, p=0.01) and Unknown ethnicity (accumulative risk difference: 13.4% vs. 8.9%, p<0.001) (Figure 1 & Table 3).

Inequalities in the percentage of unvaccinated young women by deprivation were attenuated by each stage of implementation of the policy. At Stage One (parental written consent only), 14.2% young women in the least deprived quintile were unvaccinated in comparison to 29.8% of young women in the most deprived quintile. Subsequent to Stage Two (parental verbal consent and adolescent self-consent), the percentage of unvaccinated young women decreased at a greater rate for those belonging to the most deprived quintile compared to the least deprived quintile (risk difference: 7.4% vs. 2.3%, p<0.001). A similar pattern was observed following implementation of Stage Three (community catch-up clinics and family practice settings) (overall risk difference: 13.8% vs. 5.8%, p<0.001) (Figure 1 & Table 3).

DISCUSSION

The HPV vaccination programme has been implemented to prevent HPV, a recognised precursor to developing cervical cancer. The findings from this study show that some of the barriers to young women being vaccinated were overcome through the implementation of a new local policy, which included parental verbal consent and adolescent self-consent in the school setting. There is promising evidence that the additional steps have the potential to reduce existing inequalities in uptake among young

For peer review only - http://bmjopen.bmj.com/site/about/guidelines.xhtml

women living in more deprived areas. Importantly, this study showed that these young women are less likely to engage with consent procedures that rely on paper-based methods, are more likely to be affected by cervical cancer [15, 16], and less likely to receive the HPV vaccine [6].

The majority of young women were vaccinated in the school setting. However, this study supports the provision of the HPV vaccine in community settings, such as catch-up clinics and general practice surgeries, to help improve access to vaccination. This may benefit young women who may have had anxieties about being vaccinated without a parent present, or did not attend school on the day of the vaccination session.

Community provision of the HPV vaccine also appeared to reduce substantial inequalities in uptake among young women educated in alternative education provider settings. The reasons for this are unclear, but could relate to their lower school attendance, complex behavioural and physical health needs, or perceptions of safety of vaccination in the school setting due to interactions with other medical treatment. Although they comprise a small proportion of the overall vaccine-eligible population, they are a vulnerable population with substantially lower uptake and greater health inequalities which requires addressing.

As the majority of parents who could be contacted provided verbal consent, the data imply that the absence of a signed parental consent form cannot be assumed to mean the parent does not want their daughter to have the vaccine. Not all parents could be contacted by the immunisation team on the day of the vaccination session. Provision of additional resources to contact families ahead of the vaccination session could help reduce the proportion of families who are not contacted and help ensure that their daughter receives the HPV vaccine if they wish.

Relative to parental verbal consent, adolescent self-consent occurred infrequently. Our analyses of qualitative data from this study showed a strong presumption that parents should make decisions affecting the health of their children. The preferred age at which the HPV vaccination is administered (12-13 years) also contributed to reluctance in endorsing self-consent which was thought to have the potential to break down trust between parents and school staff, and within families [17]. This suggests

For peer review only - http://bmjopen.bmj.com/site/about/guidelines.xhtml

that unresolved issues could act as a barrier to widespread implementation of adolescent self-consent in other settings.

Our primary statistical analyses for this study showed that the new consent procedures increased uptake by 11% in one of the intervention local authorities, and appeared to overcome trends for decreasing uptake in matched sites [11]. Our secondary analyses of the process evaluation data reported here showed an additional 347 young women (4.8%) received the HPV vaccine in the school setting. All things being equal, if changes in policy resulted in similar effect sizes in other local authorities, as part of a strategy to increase uptake, then the English HPV vaccination programme could reach the World Health Organisation's target of 90% of young women receiving the vaccination by 15 years old [18].

Additionally, establishing the cost-effectiveness of strategies to improve uptake of vaccination programmes is important to provide evidence for policy makers to target resources appropriately. This has been established in the context of the United States of America [19], but these findings are not easily translatable to English schools-based, rather than healthcare-based, vaccination programmes.

Public Health England have recently issued updated guidance for healthcare professionals related to the new universal HPV vaccination programme [20]. This supports the use of parent verbal consent and adolescent self-consent as strategies to maximise uptake and reduce catch-up sessions. They cite further benefit of inclusion of young people whose parents may have difficulties in completing the consent due to language or literacy issues. The findings from this study provide evidence that strategies incorporating parent verbal consent could help young women belonging to 'harder-to-reach' families receive the HPV vaccine. These recommendations may also be applicable to other schools-based vaccination programmes, including the influenza vaccination programme offered to primary school aged children where similar patterns in forms returns have been reported [21].

Strengths and limitations

The study has some strengths. This is the first study to examine how new local polices for the HPV vaccination programme are implemented, and the impact on health inequalities among more deprived

For peer review only - http://bmjopen.bmj.com/site/about/guidelines.xhtml

populations and young women belonging to minority ethnicity groups. Our study utilised routinely collected data related to vaccination status, eliminating the risk of recall and selection bias. The data relate to vaccinations delivered in school and community settings to all young women eligible for routine HPV vaccination during the study period. As such, our results correspond to an almost complete population.

There are some limitations. The data related to implementation of new consent procedures in a geographically distinct area in the south-west of England. The findings therefore may not be applicable to local authorities that are implementing new consent procedures in schools-based vaccination programmes elsewhere in the United Kingdom. The findings may also not translate to other adolescent vaccination programmes delivered in countries where cultural differences may influence the acceptability of parental verbal consent and adolescent self-consent procedures in the school setting.

As the study relied on routinely collected information, we did not have access to individual-level measures of socioeconomic status and relied on area-based measures of deprivation. Our study findings may therefore be subject to ecological fallacy.

An issue, common to all routinely collected data, is the possibility of data input errors and missing data. To minimise bias from inclusion of this data, we excluded almost 5% of the data as the information related to school was out-of-date. Overall, our dataset identified 8% of young women belonging to a minority ethnic group. This compares with nationally reported figures indicating 30% of young people attending secondary schools in the intervention areas belong to a non-White ethnic group [22]. Missing ethnicity data (23%) relating to young women who were born outside the local authority boundaries could change the direction or size of aORs corresponding to ethnicity.

Conclusions

Introducing further steps to the consent procedures – allowing parents to consent verbally and adolescent self-consent – overcame some of the barriers to vaccination of young women belonging to families less likely to respond to paper-based methods of gaining consent.

For peer review only - http://bmjopen.bmj.com/site/about/guidelines.xhtml

REFERENCES

1. Drolet, M., et al., Population-level impact and herd effects following the introduction of human papillomavirus vaccination programmes: updated systematic review and meta-analysis. The Lancet, 2019. **394**(10197): p. 497-509.

2. Simms, K.T., et al., Impact of scaled up human papillomavirus vaccination and cervical screening and the potential for global elimination of cervical cancer in 181 countries, 2020;99: a modelling study. The Lancet Oncology, 2019. **20**(3): p. 394-407.

3. Johnson, H.C., et al., Effect of HPV vaccination and cervical cancer screening in England by ethnicity: a modelling study. The Lancet Public Health, 2018. **3**(1): p. e44-e51.

4. Baker, D. and E. Middleton, Cervical screening and health inequality in England in the 1990s. Journal of Epidemiology and Community Health 2003. **57**(6): p. 417-423.

5. Shack, L., et al., Variation in incidence of breast, lung and cervical cancer and malignant melanoma of skin by socioeconomic group in England. BMC Cancer, 2008. **8**(1): p. 271.

6. Fisher, H., et al., Examining inequalities in the uptake of the school-based HPV vaccination programme in England: a retrospective cohort study. Journal of Public Health, 2013. **36**(1): p. 36-45.

7. Batista Ferrer, H., et al., Barriers and facilitators to uptake of the school-based HPV vaccination programme in an ethnically diverse group of young women. Journal of Public Health, 2016. **38**(3): p. 569-577.

8. Batista Ferrer, H., Factors influencing the uptake of the Human Papillomavirus (HPV) vaccination programme. Thesis (Ph.D.), 2014.

9. Gillick V . West Norfolk and Wisbech Area Health Authority and Department of Health and Social Security. 1985, House of Lords: London.

10. Fisher, H., et al., Barriers and enablers to adolescent self-consent for vaccination: A mixed-methods evidence synthesis. Vaccine, 2019. **37**(3): p. 417-429.

11. Fisher, H., et al., Impact of new consent procedures on uptake of the schools-based human papillomavirus (HPV) vaccination programme. Journal of Public Health, 2020.

12. Audrey, S., et al., Impact and acceptability of self-consent procedures for the school-based human papillomavirus vaccine: a mixed-methods study protocol. BMJ Open. 2018. **8**(3): p. e021321.

13. English indices of deprivation, 2019. Available from: https://www.gov.uk/government/statistics/english-indices-of-deprivation-2019.

14. Hughes, R.A., et al., Accounting for missing data in statistical analyses: multiple imputation is not always the answer. International Journal of Epidemiology, 2019. **48**(4): p. 1294-1304.

15. Shack, L., et al., Variation in incidence of breast, lung and cervical cancer and malignant melanoma of skin by socioeconomic group in England. BMC Cancer, 2008. **8**: p. 271.

16. Cancer Research UK and National Cancer Intelligence Network. Cancer by deprivation in England: Incidence, 1996-2010, Mortality, 1997-2011. 2014, NCIN: London.

17. Audrey, S., et al., How acceptable is adolescent self-consent for the HPV vaccination: qualitative findings from a process evaluation in south-west England. Vaccine, 2020. https://doi.org/10.1016/j.vaccine.2020.09.074.

18. World Health Organisation, Strategic Advisory Group of Experts on Immunization: Working Group on Human Papillomavirus Immunization, 2018. Available from:

For peer review only

https://www.who.int/immunization/sage/meetings/2018/october/3_SAGE2018_WG_recommendation_FINAL.pdf.

19. Spencer, J.C., et al., Cost-effectiveness of Interventions to Increase HPV Vaccine Uptake. Pediatrics. 2020. **146**(6): p. e20200395.

20. Public Health England, HPV vaccination: Information for healthcare practitioners. 2019, Available from: https://assets.publishing.service.gov.uk/government/uploads/system/uploads/attachment_data/file/813014/PHE_HPV_universal_programme_guidance.pdf.

21. Christensen, H., et al., Influence of commissioned provider type and deprivation score on uptake of the childhood flu immunization. Journal of Public Health, 2019.

22. Department for Education, Schools, pupils and their characteristics: January 2018. Avaialble from: https://www.gov.uk/government/statistics/schools-pupils-and-their-characteristics-january-2018.

For peer review only

DECLARATIONS

Consent & ethical considerations

Research Ethics Committee approval is not required for evaluations that are designed solely to define or judge current care and measure a current service without reference to a standard. This evaluation involves the retrospective analysis of an anonymised dataset comprising data routinely collated by the immunisation teams and the Child Health Information Services as part of the HPV vaccination programme. The study did not involve the collection of additional data. Research Ethics Committee approval was therefore not required. However, permission for the research team at the University of Bristol to receive an anonymised dataset was sought from the organisations assigned as data custodians for the data (Sirona Health and Care and In Health Intelligence).

Patient consent

Not required.

Data sharing statement

As the analysis was undertaken on routine data acquired from an external source, the study authors assured the data custodians that the dataset would be treated as confidential and would not be shared.

Competing interests

The authors have no competing interests to declare.

For peer review only - http://bmjopen.bmj.com/site/about/guidelines.xhtml

Funding

This work is funded by the National Institute for Health Research's Research for Patient Benefit (NIHR RfPB) programme (project number PB-PG-0416-20013). The views and opinions expressed therein are those of the authors and do not necessarily reflect those of the NIHR RfPB Programme or the Department of Health.

Contributorship statement

SA, HF, MH, KE, JF & MR were involved in the conception and design of the research. SA is principal investigator; HF is study manager and undertook data cleaning, analysed the data, and drafted the manuscript; JF, JY and KE developed and advised on the new consent procedures and the HPV vaccination process; MR advised on local immunisation strategy; JM advised on health inequalities and MH and RR advised on statistical methods. All authors have made substantial contributions to interpreting the data, revising it for important intellectual content, and have given approval of the final version to be submitted.

Acknowledgements

The authors express their gratitude to staff at In Health Intelligence for data linkage and extracting the data from the Child Health Information System, and to the immunisation and administrative teams at Sirona Healthcare who facilitated access to the immunisation consent records as part of this study.

The study is partly supported by the NIHR Health Protection Research Unit in Evaluation of Interventions at University of Bristol. This research was supported by the National Institute for Health Research (NIHR) Applied Research Collaboration West (NIHR ARC West). The views expressed in this article are those of the author(s) and not necessarily those of the NIHR or the Department of Health and Social Care. The work was undertaken with the support of The Centre for the Development and Evaluation of Complex Interventions for Public Health Improvement (DECIPHer), a UKCRC Public Health Research Centre of Excellence. Joint funding (MR/KO232331/1) from the British Heart Foundation, Cancer Research UK, Economic and Social Research Council, Medical Research Council,

For peer review only - http://bmjopen.bmj.com/site/about/guidelines.xhtml

the Welsh Government and the Wellcome Trust, under the auspices of the UK Clinical Research Collaboration, is gratefully acknowledged.

For peer review only

For peer review only - http://bmjopen.bmj.com/site/about/guidelines.xhtml

Table 1. Descriptive summary of eligible cohort by vaccine receipt and return of parent consent form

	**Eligible cohort n (%)**	**HPV vaccine received n (%)**	**Unreturned parent consent form n (%)**
	7,129 (100.0)	6,341 (89.0)	1,155 (16.2)
Area-level			
Local Authority One	4,516 (63.4)	3,944 (87.3)	843 (18.7)
Local Authority Two	2,613 (36.7)	2,397 (91.7)	312 (11.9)
Programme year 2017/18	3,581 (50.2)	3,202 (89.4)	565 (15.8)
Programme year 2018/19	3,548 (49.8)	3,139 (88.5)	590 (16.6)
School category			
Comprehensive, non-fee-paying	6,350 (89.1)	5,690 (89.6)	992 (15.6)
Private, fee-paying	661 (9.3)	565 (85.5)	105 (15.9)
Alternative education providers	118 (1.7)	86 (72.9)	58 (49.2)
Individual-level			
Ethnicity			
White British	4,888 (68.6)	4,552 (93.1)	610 (12.5)
Non-White British	572 (8.0)	492 (86.0)	101 (17.7)
Unknown	1,669 (23.4)	1,297 (77.7)	444 (26.6)
Deprivation			
Least deprived	1,348 (18.9)	1,235 (91.6)	136 (10.1)
Quintile 2	1,379 (19.3)	1,277 (92.6)	149 (10.8)
Quintile 3	1,403 (19.7)	1,273 (90.7)	210 (15.0)
Quintile 4	1,396 (19.6)	1,203 (86.2)	292 (20.9)
Most deprived	1,421 (19.9)	1,194 (84.0)	338 (23.8)
Unknown	182 (3.6)	159 (87.4)	30 (16.5)

For peer review only

For peer review only - http://bmjopen.bmj.com/site/about/guidelines.xhtml

Table 2. Associations of unreturned parental consent form with school category, ethnicity & deprivation

	N (%)	Form not returned	OR (95% CI) †	p-value	aOR (95% CI) †	p-value
			-	-	-	-
School category		**n (%)**				
Comprehensive, non-fee-paying	6,350	992 (15.6)	-	-		
Private, fee-paying	661	105 (15.9)	1.02 (0.82-1.27)	0.86	1.12 (0.89-1.43)	0.34
Alternative education providers	118	58 (49.2)	5.22 (3.62-7.54)	<0.001	5.54 (3.80-8.09)	<0.001
Ethnicity						
White British	4,888	610 (12.5)	-	-	-	-
Non-White British	572	101 (17.7)	1.50 (1.19-1.89)	<0.01	1.34 (1.06-1.70)	0.01
Unknown	1,669	444 (26.6)	2.54 (2.21-2.92)	<0.001	2.41 (2.09-2.78)	<0.001
Deprivation						
Least deprived	1,279	136 (10.1)	-		-	
Quintile 2	1,324	149 (10.8)	1.08 (0.84-1.38)	0.54	1.09 (0.85-1.40)	0.48
Quintile 3	1,334	210 (15.0)	1.57 (1.25-1.97)	<0.001	1.57 (1.24-1.98)	<0.001
Quintile 4	1,305	292 (20.9)	2.36 (1.89-2.93)	<0.001	2.24 (1.79-2.81)	<0.001
Most deprived	1,347	338 (23.8)	2.78 (2.24-3.45)	<0.001	2.54 (2.03-3.18)	<0.001
Unknown	182	30 (16.4)	1.75 (1.14-2.70)	0.01	1.52 (0.97-2.37)	0.07

† Adjusted for ethnicity, deprivation, school category, and clustering by school; OR: Odds Ratio; CI: Confidence Intervals; aOR: Adjusted Odds Ratio;

For peer review only

Table 3. Percentage of young women unvaccinated by implementation stage of consent process

		Stage 1†	Stages 1 & 2	Risk reduction[1]	p-value[1]	Stages 1, 2 & 3	Risk reduction[2]	p-value[2]
		n (%)	n (%)	n (%)		n (%)	n (%)	-
		1,519 (21.3)	1,173 (16.5)	347 (4.8)		788 (11.1)	731 (10.3)	
School category	**N (%)**	**% (95% CI)**	**% (95% CI)**	**% (95% CI)**	**-**	**% (95% CI)**	**% (95% CI)**	
Comprehensive, non-fee-paying	6,350	20.6 (19.6-21.6)	15.4 (14.5-16.3)	5.3 (4.7-5.8)	-	10.4 (9.7-11.2)	10.2 (9.5-11.0)	-
Private, fee-paying	661	22.7 (19.7-26.0)	21.5 (18.5-24.8)	1.2 (0.6-2.4)	<0.001	14.5 (12.0-17.4)	8.2 (6.3-10.5)	0.07
Alternative education providers	118	50.8 (41.9-59.7)	47.5 (38.7-56.4)	3.4 (1.3-8.7)	0.27	27.1 (19.9-35.8)	23.7 (16.9-32.3)	<0.001
Ethnicity								
White British	4,705	15.8 (14.8-16.8)	11.6 (10.7-12.5)	4.2 (3.6-4.8)	-	6.9 (6.2-7.6)	8.9 (8.1-9.7)	-
Non-White British	542	26.7 (23.3-30.5)	21.3 (18.2-24.9)	5.4 (3.8-7.6)	0.21	14.0 (11.4-17.1)	12.8 (10.3-15.8)	0.01
Unknown	1,503	35.7 (33.4-38.0)	29.0 (26.9-31.2)	6.7 (5.6-8.0)	<0.001	22.3 (20.4-24.3)	13.4 (11.8-15.1)	<0.001
Deprivation								
Least deprived	1,279	14.2 (12.4-16.1)	11.9 (10.3-13.7)	2.3 (1.6-3.3)	-	8.4 (7.0-10.0)	5.8 (4.7-7.2)	-
Quintile 2	1,324	14.9 (13.1-16.8)	11.2 (9.7-13.0)	3.6 (2.8-4.8)	0.04	7.4 (6.1-8.9)	7.5 (6.2-9.0)	0.08
Quintile 3	1,334	19.8 (17.8-22.0)	14.9 (13.1-16.9)	4.9 (3.9-6.2)	<0.001	9.3 (7.9-10.9)	10.5 (9.0-12.3)	<0.001
Quintile 4	1,305	27.1 (24.8-29.5)	20.8 (18.8-23.1)	6.2 (5.1-7.6)	<0.001	13.8 (12.1-15.7)	13.3 (11.6-15.1)	<0.001
Most deprived	1,347	29.8 (27.4-32.2)	22.4 (20.3-24.6)	7.4 (6.1-8.9)	<0.001	16.0 (14.2-18.0)	13.8 (12.1-15.7)	<0.001
Unknown		24.2 (18.5-30.9)	22.0 (16.6-28.5)	12.6 (8.6-18.2)	0.93	12.6 (8.5-18.3)	11.5 (7.6-17.1)	0.02

† Stage 1: Parental written consent only; Stage 2: Schools-based procedures (parental verbal consent & adolescent self-consent; Stage 3: Community settings (catch-up clinics & family practices); CI: Confidence intervals;

[1]Risk difference for stages 1 & 2 compared to stage 1; p-value for comparison with risk difference in baseline group.

[2]Risk difference for stages 1 & 2 & 3 compared to stage 1; p-value for comparison with risk difference in baseline group.

For peer review only

For peer review only - http://bmjopen.bmj.com/site/about/guidelines.xhtml

Figure legends

Figure 1. Percentage of HPV vaccine eligible young women unvaccinated by stage of implementation of consent procedure

For peer review only

For peer review only - http://bmjopen.bmj.com/site/about/guidelines.xhtml

Figure 1. Percentage of HPV vaccine eligible young women unvaccinated by stage of implementation of consent procedure

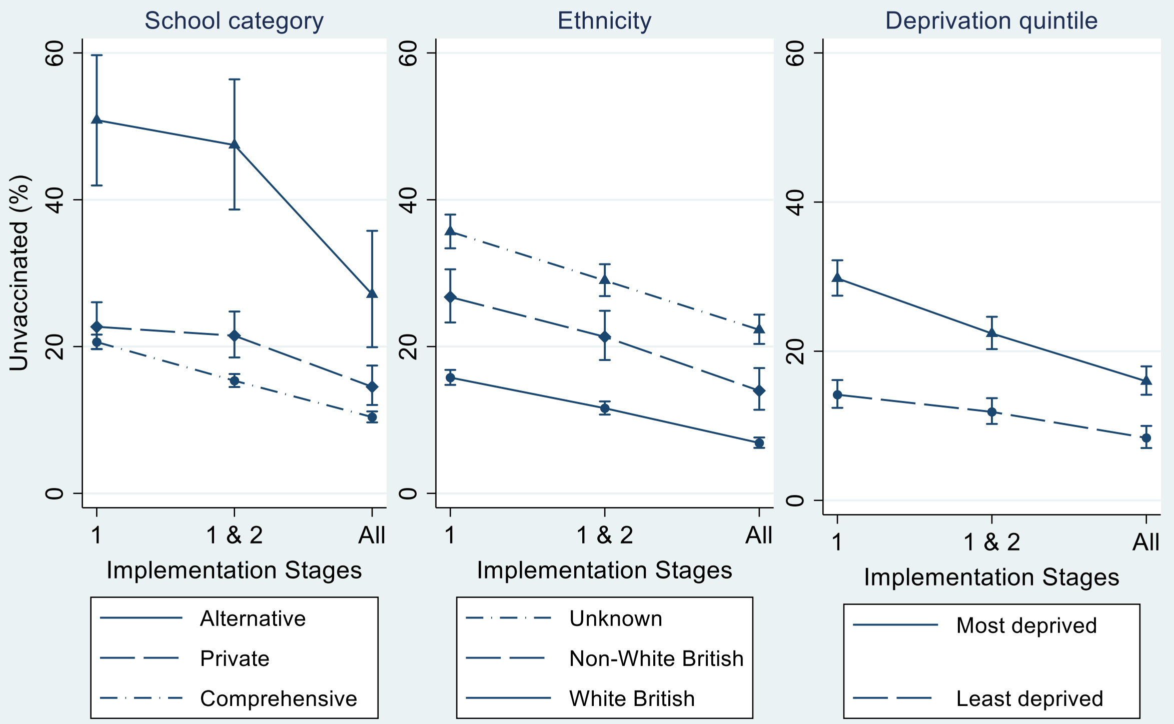

Stage of implementation
Stage 1: Parental written consent only
Stage 2: Schools-based procedures (parental verbal consent & adolescent self-consent)
Stage 3: Community settings (catch-up clinics & family practices)

Supplementary material 1. Pathway of new consent procedures

Stage One

Parental written consent

- Consent forms sent home via school to parents
- School collate forms for the vaccination session
- Young women with signed consent forms receive the HPV vaccine

Stage Two

Parental verbal consent & adolescent self-consent

- For young women without signed consent forms, parents telephoned for opportunity to verbally consent
- Young women asked whether discussion about vaccination had taken place at home
- Young women assessed for self-consent by immunisation team

Stage Three

Community catch-up clinics & family practice

- Unvaccinated young women are provided with written information about community catch-up clinics
- Some young women may also choose to be vaccinated in the family practice setting

For peer review only

STROBE Statement—Checklist of items that should be included in reports of ***cross-sectional studies***

	Item No	Recommendation	Page No
**Title and abstract**	1	(*a*) Indicate the study's design with a commonly used term in the title or the abstract	3
		(*b*) Provide in the abstract an informative and balanced summary of what was done and what was found	3
**Introduction**			
Background/rationale	2	Explain the scientific background and rationale for the investigation being reported	4-5
Objectives	3	State specific objectives, including any prespecified hypotheses	5
**Methods**			
Study design	4	Present key elements of study design early in the paper	5
Setting	5	Describe the setting, locations, and relevant dates, including periods of recruitment, exposure, follow-up, and data collection	6-8
Participants	6	(*a*) Give the eligibility criteria, and the sources and methods of selection of participants	6-8
Variables	7	Clearly define all outcomes, exposures, predictors, potential confounders, and effect modifiers. Give diagnostic criteria, if applicable	6-8
Data sources/ measurement	8*	For each variable of interest, give sources of data and details of methods of assessment (measurement). Describe comparability of assessment methods if there is more than one group	6-8
Bias	9	Describe any efforts to address potential sources of bias	7
Study size	10	Explain how the study size was arrived at	7-9
Quantitative variables	11	Explain how quantitative variables were handled in the analyses. If applicable, describe which groupings were chosen and why	7-8
Statistical methods	12	(*a*) Describe all statistical methods, including those used to control for confounding	8-9
		(*b*) Describe any methods used to examine subgroups and interactions	8-9
		(*c*) Explain how missing data were addressed	7
		(*d*) If applicable, describe analytical methods taking account of sampling strategy	N/A
		(*e*) Describe any sensitivity analyses	N/A
**Results**			
Participants	13*	(a) Report numbers of individuals at each stage of study—eg numbers potentially eligible, examined for eligibility, confirmed eligible, included in the study, completing follow-up, and analysed	9
		(b) Give reasons for non-participation at each stage	9
		(c) Consider use of a flow diagram	N/A
Descriptive data	14*	(a) Give characteristics of study participants (eg demographic, clinical, social) and information on exposures and potential confounders	9
		(b) Indicate number of participants with missing data for each variable of interest	9, 17
Outcome data	15*	Report numbers of outcome events or summary measures	N/A
Main results	16	(*a*) Give unadjusted estimates and, if applicable, confounder-adjusted estimates and their precision (eg, 95% confidence interval). Make clear which confounders were adjusted for and why they were included	9-11, 18-19

For peer review only - http://bmjopen.bmj.com/site/about/guidelines.xhtml
For peer review only

		(*b*) Report category boundaries when continuous variables were categorized	
		(*c*) If relevant, consider translating estimates of relative risk into absolute risk for a meaningful time period	
Other analyses	17	Report other analyses done—eg analyses of subgroups and interactions, and sensitivity analyses	N/A
**Discussion**			
Key results	18	Summarise key results with reference to study objectives	11
Limitations	19	Discuss limitations of the study, taking into account sources of potential bias or imprecision. Discuss both direction and magnitude of any potential bias	12-13
Interpretation	20	Give a cautious overall interpretation of results considering objectives, limitations, multiplicity of analyses, results from similar studies, and other relevant evidence	13
Generalisability	21	Discuss the generalisability (external validity) of the study results	13
**Other information**			
Funding	22	Give the source of funding and the role of the funders for the present study and, if applicable, for the original study on which the present article is based	15

*Give information separately for exposed and unexposed groups.

Note: An Explanation and Elaboration article discusses each checklist item and gives methodological background and published examples of transparent reporting. The STROBE checklist is best used in conjunction with this article (freely available on the Web sites of PLoS Medicine at http://www.plosmedicine.org/, Annals of Internal Medicine at http://www.annals.org/, and Epidemiology at http://www.epidem.com/). Information on the STROBE Initiative is available at www.strobe-statement.org.

For peer review only
